# Comprehensive optimization of a reporter assay toolbox for three distinct CRISPR‐Cas systems

**DOI:** 10.1002/2211-5463.13198

**Published:** 2021-06-09

**Authors:** Li Chen, Haoyuan Gao, Bing Zhou, Yu Wang

**Affiliations:** ^1^ State Key Laboratory of Stem Cell and Reproductive Biology Institute of Zoology Chinese Academy of Sciences Beijing China; ^2^ University of Chinese Academy of Sciences Beijing China; ^3^ College of Life Sciences and Oceanography Shenzhen University China; ^4^ Department of Biology Oberlin College OH USA; ^5^ Institute for Stem Cell and Regeneration Chinese Academy of Sciences Beijing China

**Keywords:** CRISPR, DNA repair, gene editing, reporter assay, small molecules

## Abstract

The clustered, regularly interspaced, short palindromic repeats‐associated DNA nuclease (CRISPR‐Cas) protein system allows programmable gene editing through inducing double‐strand breaks. Reporter assays for DNA cleavage and DNA repair events play an important role in advancing the CRISPR technology and improving our understanding of the underlying molecular mechanisms. Here, we developed a series of reporter assays to probe mechanisms of action of various editing processes, including nonhomologous DNA end joining, homology‐directed repair and single‐strand annealing. With special target design, the reporter assays as an optimized toolbox can be used to take advantage of three distinct CRISPR‐Cas systems (*Streptococcus pyogenes* Cas9, *Staphylococcus aureus* Cas9 and *Francisella novicida U112* Cpf1) and two different reporters (GFP and Gaussia luciferase). We further validated the Gaussia reporter assays using a series of small molecules, including NU7441, RI‐1 and Mirin, and showcased the use of a GFP reporter assay as an effective tool for enrichment of cells with edited genome.

## Graphical abstract

We introduce a series of optimized clustered, regularly interspaced, short palindromic repeats (CRISPR) reporter assays that can use three CRISPR‐Cas systems, *Streptococcus pyogenes* Cas9, *Staphylococcus aureus* Cas9 and *Francisella novicida U112* Cpf1, for probing various gene‐editing mechanisms, including nonhomologous DNA end joining, homology‐directed repair and single‐strand annealing. The use of GFP and Gaussia luciferase as reporters supports various applications, including high‐throughput drug screening and enrichment of the edited cell population.

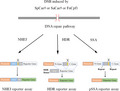

**Abbreviations**
BFP: Blue Fluorescent Protein
Cas: CRISPR‐associated DNA nuclease
CRISP: clustered, regularly interspaced, short palindromic repeats
DNA‐PKcs: DNA‐dependent protein kinase catalytic subunit
DSB: double‐strand break
FCR: Fluorescence Conversion Reporter
FnCpf1: *Francisella novicida U112* Cpf1
gRNA: guide RNA
HDR: homology‐directed repair
mG: membrane localized GFP
MRN: MRE11–RAD50–NBS1
mT: membrane localized Tomato
NHEJ: nonhomologous DNA end joining
PAM: protospacer adjacent motif
SaCas9: *Staphylococcus aureus* Cas9
SpCas9: *Streptococcus pyogenes* Cas9
SSA: single‐strand annealing
TIDE: tracking of indels by decomposition

The clustered, regularly interspaced, short palindromic repeats‐associated DNA nuclease (CRISPR‐Cas) protein system introduces specific DNA cleavages and gene editing in a programmable sequence‐dependent manner [1, 2]. The match between guide RNA (gRNA) and target DNA sequence adjacent with protospacer adjacent motif (PAM) enables the corresponding CRISPR‐associated DNA nuclease (Cas) to cleave the target site [1, 2]. Three prevailing CRISPR‐Cas systems are used in this study, including *Streptococcus pyogenes* Cas9 (SpCas9), *Staphylococcus aureus* Cas9 (SaCas9), and *Francisella novicida U112* Cpf1 (FnCpf1; Fig. 1). SpCas9 recognizes a 5′‐NGG PAM (Fig. 1A) [1, 2], while SaCas9 recognizes a 5′‐NNGRRT PAM (Fig. 1B) [3]. They belong to the Cas9 family and result in blunt‐end cleavages upstream of the PAM [1, 2, 3]. FnCpf1, in contrast, recognizes a 5′‐TTN PAM, targets a downstream locus and results in a 5‐nt overhang (Fig. 1C) [4].

**Fig. 1:**
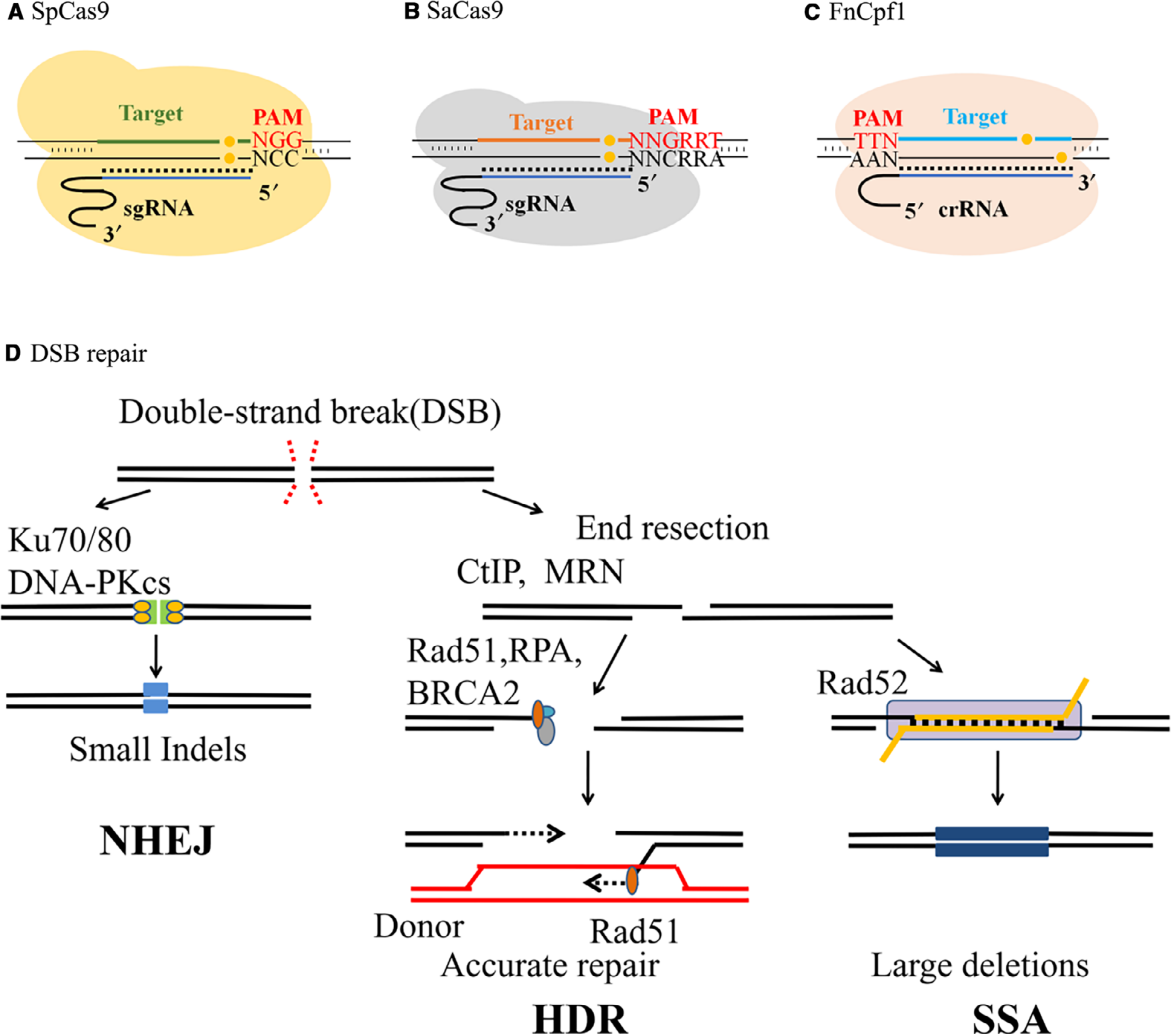
Mechanisms of CRISPR nucleases and DSB repair pathways. (A) Function principle of SpCas9‐mediated gene editing. By using a 20‐nt matching sgRNA, SpCas9 targets the specific locus (shown in green) upstream a 5′‐NGG PAM (shown in red) and results in a blunt DSB (shown in yellow) 3 bp prior to PAM. (B) Function principle of SaCas9‐mediated gene editing. By using a 20‐ or 21‐nt matching sgRNA, SaCas9 targets the specific locus (shown in orange) upstream a 5′‐NNGRRT PAM (shown in red) and results in a blunt DSB (shown in yellow) 3 bp prior to PAM. (C) Function principle of FnCpf1‐mediated gene editing. By using a 23‐nt CRISPR RNA (crRNA), FnCpf1 targets the specific locus (shown in blue) downstream a 5′‐TTN PAM (shown in red) and results in a 5‐nt 5′ overhang DSB (shown in yellow). (D) DSB repair pathways. The repair choices of DSBs rely primarily on whether DNA end resection occurs and whether homologous sequence is present. When resection is blocked by Ku70/80 and DNA PKcs, repair through NHEJ is favored. When DNA resection occurs and with the presence of homologous sequences, HDR and SSA pathways can compete for the repair of DSBs. Each of the repair pathways leads to different genetic outcomes. The key factors participate in each pathway, and the fidelities of the repair mechanisms are indicated.

The CRISPR‐Cas system induces programmable gene editing through causing double‐strand breaks (DSBs) [1, 2]. Nonhomologous DNA end joining (NHEJ), homology‐directed repair (HDR) and single‐strand annealing (SSA) are the most common DSB repair pathways in mammalian cells (Fig. 1D) [5, 6, 7]. The NHEJ pathway repairs DSB with blunt ends in help with factors that protect the blunt ends from further resection, such as Ku70/80 and DNA‐dependent protein kinase catalytic subunit (DNA‐PKcs) [5]. The outcomes are insertion or deletion (indels) of small sizes at the cleavage site with high randomness [5, 6, 8, 9]. In other DSB repair mechanisms, including SSA and HDR, the MRE11–RAD50–NBS1 (MRN) complex and the CtIP protein first bind to the DSB sites and generate resected ends [5, 10]. The SSA pathway mediates DSB repair by annealing exposed complementary sequences with repeated ends and conserves only one copy of the repeat in the repaired sequence [11, 12]. Unlike SSA, HDR happens in the presence of an extrahomologous donor as the recombination template [13, 14]. Binding between the resected DSB ends and the Rad51 protein enables the donor to perform homologous recombination at the break site [5, 15, 16]. In contrast with NHEJ, SSA and HDR provide error‐free DNA repair with high specificity (Fig. 1D) [16].

As one of the most powerful gene‐editing tools, CRISPR technology is in demand of complementary reporter assay systems to precisely and effectively detect post‐CRISPR editing events. Previous reporter assays based on the I‐*Sce*I endonuclease, the frequently used method for generating DNA DSBs before the advent of CRISPR technology, were developed to probe distinct DNA repair mechanisms [17, 18, 19, 20, 21]. Many of the I‐*Sce*I reporter assays were further modified and applied to the CRISPR‐SpCas9 system, including Direct Repair‐GFP Reporter and Traffic Light Reporter, since the CRISPR technology became dominant in the field of gene editing [22, 23, 24, 25, 26]. Researchers have also developed CRISPR reporter assays, such as end‐joining GFP reporter and CRISPR‐Cas based Dual‐fluorescent DSB Repair reporter [27, 28, 29]. They have greatly facilitated better understanding and advancement of the CRISPR technology [23, 24, 28, 30, 31, 32]. However, these previous reporter assays still have room for improvement in flexibility and efficiency (Table S1).

An optimized toolbox of various reporter assays derived from a comprehensive survey would facilitate the development and use of the CRISPR technology, as well as the investigations of DNA repair. To this end, we first constructed a series of reporter assays with a shared gRNA target for three CRISPR‐Cas systems, SpCas9, SaCas9 and FnCpf1, using GFP as the common fluorescent reporter. Each reporter assay can probe a specific DNA repair mechanism, including NHEJ, HDR and SSA. To expand the use of reporter assays to high‐throughput applications, we further designed a series of Gaussia luciferase‐based reporter assays and validated them with small‐molecule regulators, including NU7441, RI‐1 and Mirin [33, 34, 35, 36, 37]. Lastly, we demonstrated the pSSA‐GFP reporter assay as a tool for efficient enrichment of cells with edited genome.

## Materials and methods

### Plasmid construction

SpCas9 was cloned from the pX330‐U6‐Chimeric_BB‐CBhhSpCas9 plasmid (a gift from Feng Zhang, Addgene plasmid #422302) [2]. SaCas9 was cloned from the pX601‐AAV‐CMV::NLS‐SaCas9‐NLS‐3xHA‐bGHpA;U6::BsaI‐sgRNA plasmid [a gift from Feng Zhang, Addgene (Watertown, MA, USA) plasmid #61591] [3]. FnCpf1 was cloned from pcDNA3.1‐hFnCpf1 (a gift from Feng Zhang, Addgene plasmid #69976) [4]. Plasmids that can express both the CRISPR‐Cas endonuclease and gRNA were used to simplify the transfection system in each comparison. Reporter assay plasmids were generated by using digestion and ligation method. Reporter genes were obtained by PCR with templates containing entire GFP or Gaussia coding sequence. Sequences of each reporter assay and corresponding primers are listed in the Supporting Information (Appendices S1 and S2).

### Cell culture and transfection

HEK293T cells (ATCC, Manassas, VA, USA) were maintained in Dulbecco's modified Eagle's Medium supplemented with 10% FBS, 2 mm GlutaMAX (Thermo Fisher, Shanghai, China), 100 U·mL^−1^ penicillin and 100 μg·mL^−1^ streptomycin under 37 °C, 5% CO_2_. Transfections were done using PEI (Polysciences, Warrington, PA, USA) according to the manufacturer's recommended protocol.

In GFP reporter assays, before transfection, 150 000 cells were seeded per well in a 24‐well dish. A total of 800 ng total DNA was transfected per well in a 24‐well dish. Each construct was transfected with the same amount of substance. Five hours after transfection, fresh medium was changed.

In Gaussia reporter assays, before transfection, 25 000 cells were seeded per well in a 96‐well dish. A total of 200 ng total DNA was transfected per well in a 96‐well dish. Each construct was transfected with the same amount of substance. Five hours after transfection, fresh medium was changed.

Cells were cultured for an additional 48 h before examination.

### Small molecule treatment

After HEK293T cells were transfected, culture medium was changed 5 h after transfection with the indicated concentrations of NU7441 (CAS No. 503468‐95‐9; Selleck, Shanghai, China), RI‐1 (CAS No. 415713‐60‐9; Selleck) and Mirin (CAS No. 1198097‐97‐0; Selleck). DMSO served as a vehicle. Cells were cultured for an additional 48 h before examination.

### Flow cytometry analyses

Flow cytometry analyses of GFP‐positive cells were performed using Calibur cell analyzer (BD Coulter, Shanghai, China) to assess gene‐editing efficiency. The FITC (FL1) channel was selected without compensation. At least 10 000 cells from each sample were analyzed.

### Cell viability assay

A total of 20 000–25 000 HEK 293T cells were seeded in each well of a 96‐well dish and were cultured in indicated concentrations of small molecules. Cell numbers are consistent in each experiment. Forty‐eight hours after treatment, 10 μL cell counting solution (B34304; Bimake, Shanghai, China) was added in each well and incubated 1 h at 37 °C. The absorbance at 450 nm (*A*_450_ _nm_) was determined with a plate reader. The cell viability of wells containing DMSO‐treated cells and wells containing medium only were set, respectively, as 100% and 0%. All other viabilities were normalized to these values.

### Tracking of indels by decomposition analyses

Genomic DNA from HEK293T cells was extracted using wizard genomic DNA purification kit (Promega, Madison, WI, USA). PCR was performed to amplify target loci from 100 ng of genomic DNA using a high‐fidelity polymerase in 50‐μL reactions. The sequences of primers were listed in Table S2. The PCR products were sequenced to assess gene editing using a web tool (available at https://tide.nki.nl/) [38].

## Results

### Fluorescence Conversion Reporter assay

**Fig. 2:**
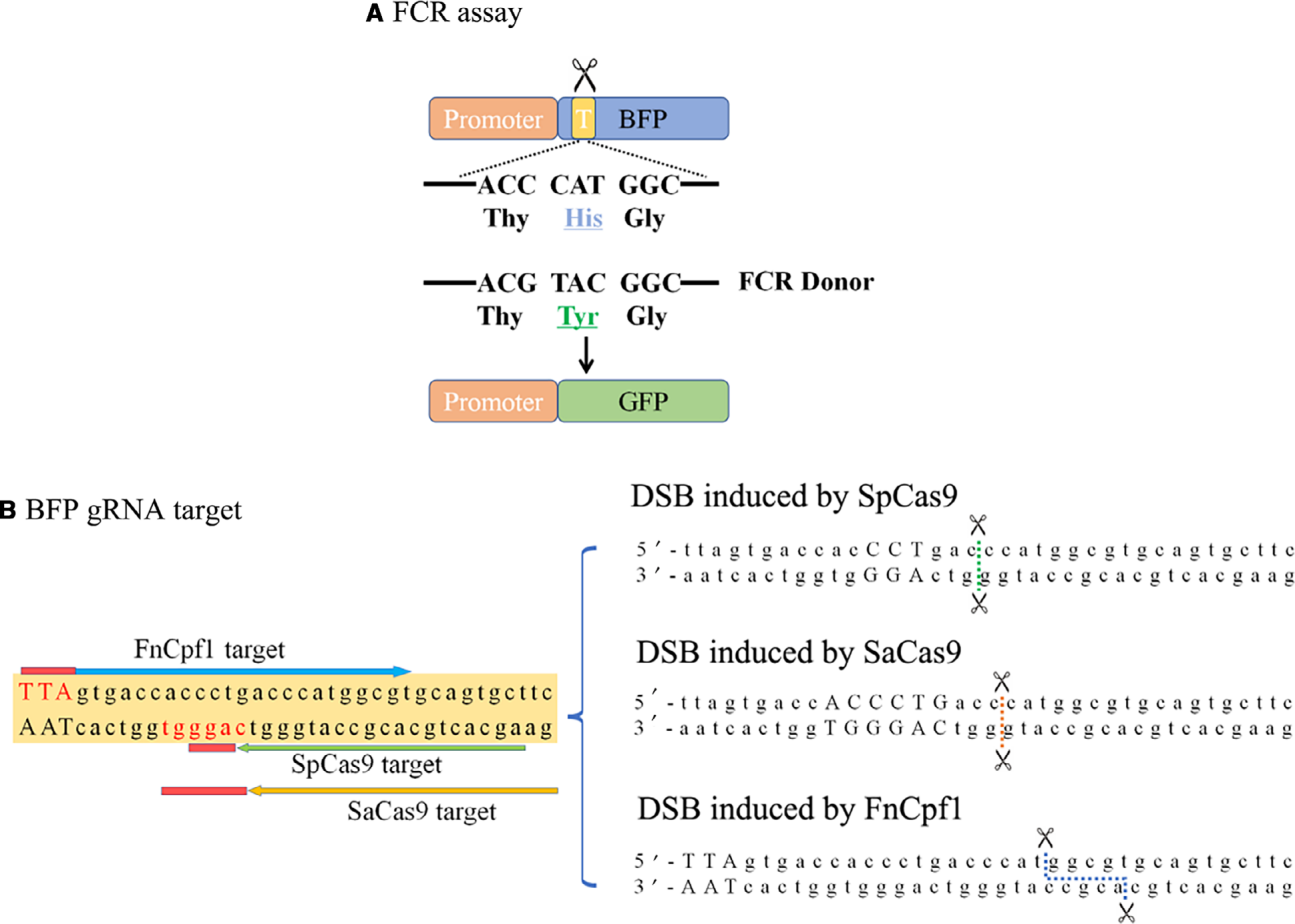
The FCR reporter assay and the BFP gRNA target. (A) Design of the FCR reporter assay. CRISPR cleavages in the target region (shown in yellow) are symbolized by the scissors. With help of the FCR donor, DSB can be repaired through the HDR pathway, changing CAT (His) to TAC (Tyr) to form a GFP reading frame (shown in green). (B) Design of the BFP gRNA target. The BFP target contains PAMs (shown in red) and gRNA targeted sites (color consistent with targets in Fig. 1A–C, respectively) for SpCas9, SaCas9 and FnCpf1. With each specific Cas, a corresponding DSB can be induced on the BFP target as shown on the right, indicated by dotted lines.

The Fluorescence Conversion Reporter (FCR) was reported to show great performance in previous studies [39, 40, 41]. FCR takes advantage of the fact that the Blue Fluorescent Protein (BFP) and the GFP share an analogous sequence and differ only in the 67th amino acid [39, 40, 41]. Therefore, with a specific codon being edited, a conversion from BFP to GFP expression is observed in the FCR reporter assay (Fig. 2A). Built from this design, by providing a TTN PAM for FnCpf1 through codon degeneracy and using the preexisting PAM for SpCas9 and SaCas9, we designed a shared gRNA target site for all three CRISPR species and named it the BFP gRNA target (Fig. 2B), which we used throughout the study.

### pSSA reporter assay

**Fig. 3:**
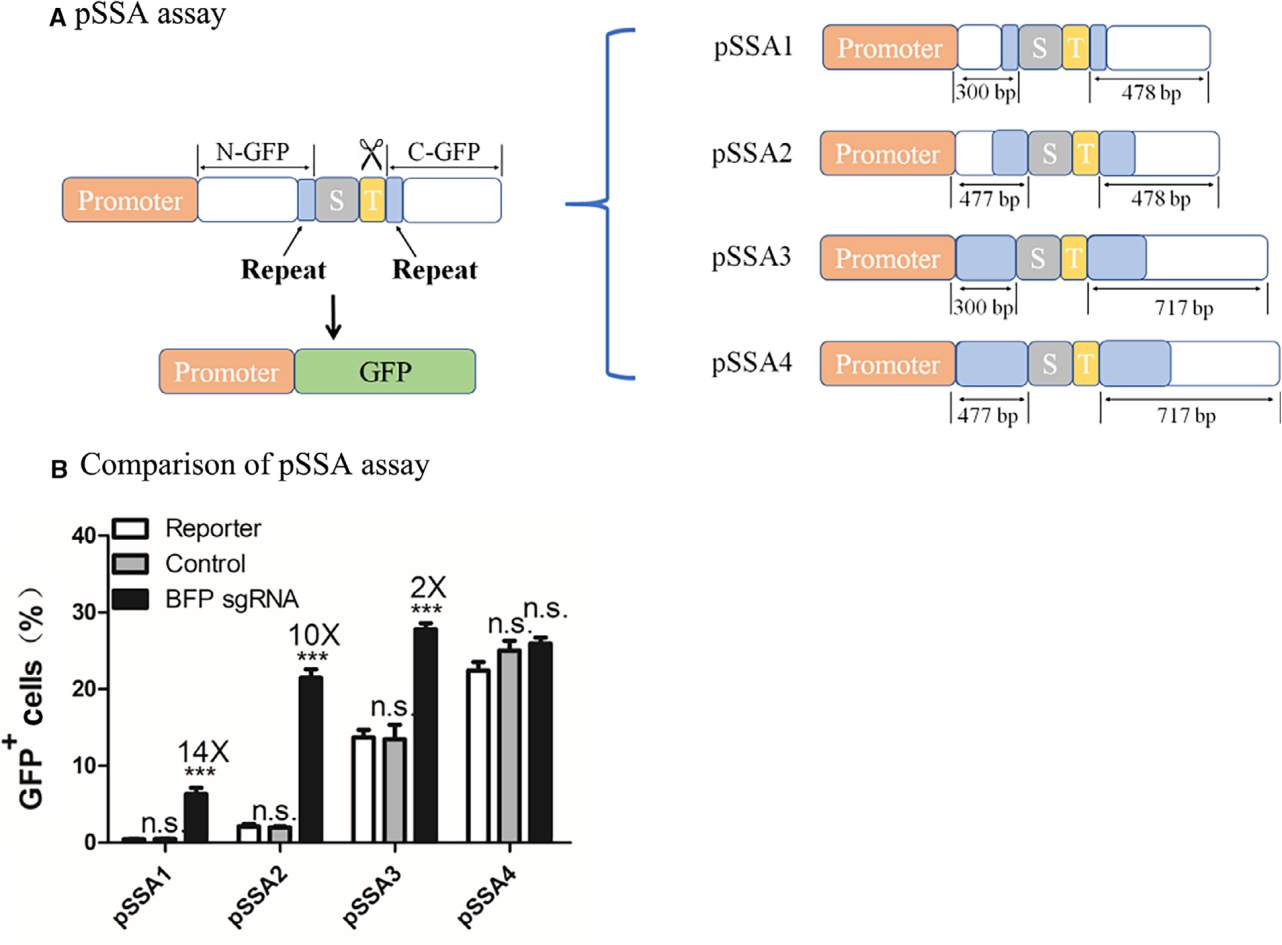
Design and optimization of the pSSA reporter assay. (A) Diagram of the pSSA reporter assay. In the pSSA assay, the two truncated GFPs (N‐GFP and C‐GFP) share a repeat sequence (shown in blue). The GFP gene is silenced (shown in white) before CRISPR‐meditated editing because of the divided N‐GFP and C‐GFP by 2× stop codons (TGATAA, shown in gray) in between. CRISPR cleavages in the target region (shown in yellow) are symbolized by the scissors. Through the SSA pathway, N‐GFP and C‐GFP are annealed together with only one copy of the repeat sequence conserved, leading to the reconstruction of a functional GFP reading frame (shown in green). On the right shows different designs of the pSSA reporter assay. The designs differ in the length of N‐GFP, C‐GFP and the repeat. The lengths of the repeat sequence (shown in blue) are continuously increasing, from 62 bp in pSSA to 1–477 bp in pSSA 4. (B) Comparison of the pSSA reporter assays. Results are obtained using SpCas9. GFP fluorescence indicates gene‐editing events. Efficiency of each pSSA assay design is quantified by counting postediting GFP‐positive cells from flow cytometry analyses. HEK293T cells are transfected with the same amount of reporter assay plasmid and SpCas9 in each comparison. The BFP sgRNA group is transfected with reporter assay plasmid and a plasmid containing SpCas9 and BFP sgRNA. The control group is transfected with reporter assay plasmid and a plasmid containing SpCas9 and scaffold sgRNA. The reporter group is transfected with reporter assay plasmid and a neutral plasmid. Data show mean ± SD. *n* = 3 biological replicates. ***P < 0.001, two‐tailed *t*‐tests. n.s., no significant difference.

In the pSSA assay, the GFP reading frame is truncated into two fragments (N‐GFP and C‐GFP in Fig. 3A) that share a repeat sequence. A stop signal is introduced between N‐GFP and the BFP gRNA target to reduce background GFP expression. After the CRISPR‐Cas system induces the cleavage at the target region, N‐GFP and C‐GFP are annealed through SSA, thus reconstituting a complete GFP reading frame [42, 43, 44, 45]. To optimize the pSSA assay for low background activity and high efficiency, we constructed a series of pSSA designs by changing the length of each fragment (Fig. 3A). The lengths of N‐GFP are 300 bp in pSSA 1 and pSSA 3 and are 477 bp in pSSA 2 and pSSA 4, while the lengths of C‐GFP are 478 bp in pSSA 1 and pSSA2 and 717 bp in pSSA 3 and pSSA 4. The lengths of repeat sequence vary in these constructs. In pSSA 3 and pSSA 4, the C‐GFP is a complete GFP coding sequence; consequently, the entire N‐GFP serves as the repeat sequence.

We used SpCas9 to examine the four pSSA reporter assay designs (Fig. 3B and Fig. S1). pSSA1 and pSSA2 both exhibit low background activity and significant efficiency. In contrast, pSSA3 and pSSA4 show high background signal, although their activities on SpCas9 transfection are also higher than pSSA1 and pSSA2. This might be because of inefficient blockade of translation by the stop signal before a complete reading frame in the C‐GFP. By comparing pSSA1 and pSSA2, longer repeat sequence appears to introduce higher efficiency (Fig. 3B). In consideration of a low background and an appropriate efficiency, we selected pSSA2 assay for further experiments, in which the repeat is one‐third of the GFP reading frame.

### HDR reporter assay

**Fig. 4:**
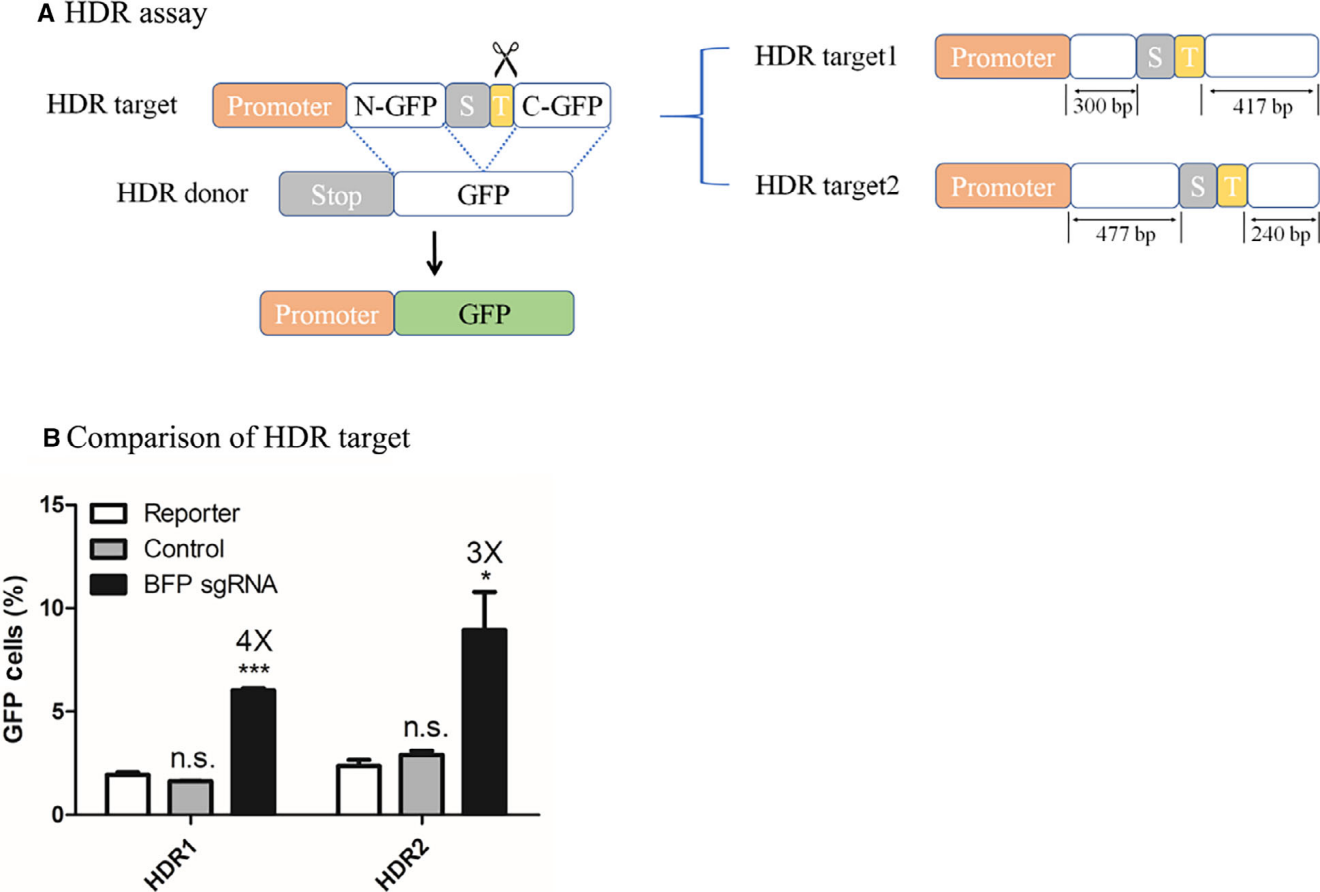
Design and optimization of the HDR reporter assay. (A) Diagram of the HDR reporter assay. In the HDR pathway, a donor is essential for DSB cleavage repair. With help of the HDR donor, two truncated GFPs (N‐GFP and C‐GFP, shown in white) in the HDR reporter assay with no overlapping sequence are replaced with a complete GFP reading frame (shown in green) through HDR. The 2× stop codons (TGATAA, shown in gray) are removed in the repaired sequence. CRISPR cleavages in the target region (shown in yellow) are symbolized by the scissors. On the right shows different designs of the HDR reporter assay, which differ in the length of N‐GFP and C‐GFP. (B) Comparison of the HDR reporter assays. Results are obtained using SpCas9. GFP fluorescence indicates gene‐editing events. Efficiency of each HDR assay design is quantified by counting postediting GFP‐positive cells from flow cytometry analyses. HEK293T cells are transfected with the same amount of reporter assay plasmid and SpCas9 in each comparison. The BFP sgRNA group contains those transfected with reporter assay plasmid and a plasmid‐containing SpCas9 and BFP sgRNA. The control group consists of those transfected with reporter assay plasmid and a plasmid containing SpCas9 and scaffold sgRNA. The reporter group contains those transfected with reporter assay plasmid and a neutral plasmid. Data show mean ± SD. *n* = 3 biological replicates. *P < 0.05; ***P < 0.001, two‐tailed *t*‐tests. n.s., no significant difference.

In the HDR reporter assay, the HDR donor contains the entire sequence of the GFP reading frame with three tandem PolyA sequences that serves as a stop signal to reduce the undesired background activity. Similar to the pSSA assays, the HDR assays contain two truncated GFP fragments named N‐GFP and C‐GFP, with a stop signal and the BFP gRNA target in between, but without repeat sequences. Taking advantage of the previous experience from pSSA assays, we avoided the use of a full GFP reading frame as C‐GFP and constructed two HDR reporter assays with different lengths of N‐GFP and C‐GFP (Fig. 4A). A GFP template, following a stop signal to reduce background noise, serves as the HDR donor for both HDR1 and HDR2. As shown in Fig. 4B and Fig. S2, HDR1 and HDR2 both have a low background and show significant GFP induction of 3‐ to 4‐fold upon SpCas9‐mediated editing. HDR2 was selected for the following experiments because of its relatively higher GFP signal.

### NHEJ reporter assay

**Fig. 5:**
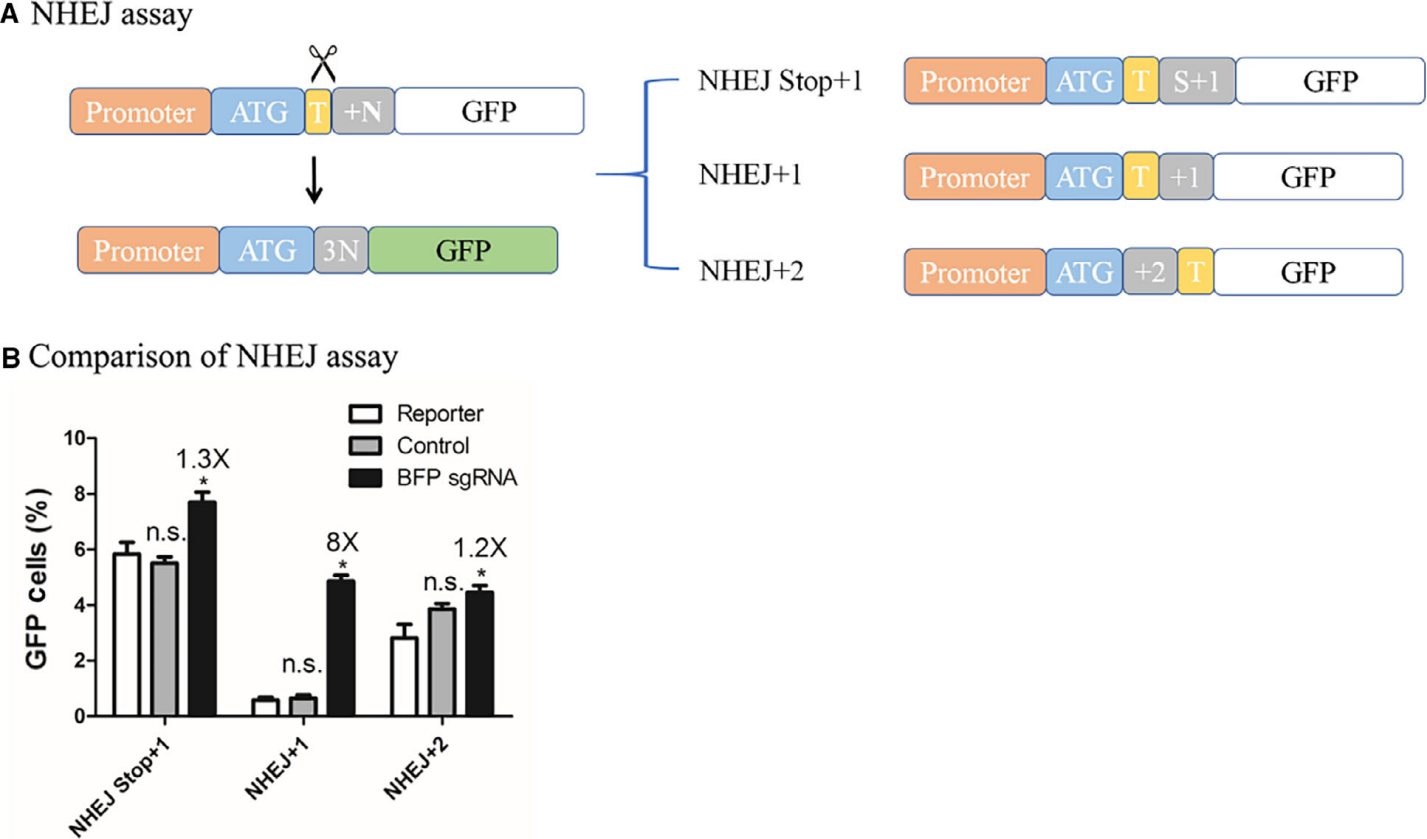
Design and optimization of the NHEJ reporter assay. (A) Diagram of the NHEJ reporter assay. The GFP gene is not expressed because of out‐of‐frame‐shift (shown in white) before CRISPR‐meditated editing. CRISPR cleavages in the target region (shown in yellow) are symbolized by the scissors. With specific NHEJ repairing events that result in a multiple of three in base pairs (3N, shown in gray) in between the promoter and the GFP gene, the GFP reading frame is corrected (shown in green). On the right shows different designs of the NHEJ reporter assay. The designs differ in the +N region (shown in gray) to probe distinct frameshifts. In NHEJ Stop + 1, the S + 1 (shown in gray) includes 3× stop codons (TAGTAGTAG) and an additional base pair. In NHEJ + 1, the +1 (shown in gray) represents a single newly introduced base pair. In NHEJ + 2, the +2 (shown in gray) contains two extra base pairs. (B) Comparison of the NHEJ reporter assays. Results are obtained using SpCas9. GFP fluorescence indicates gene‐editing events. Efficiency of each NHEJ assay design is quantified by counting postediting GFP‐positive cells from flow cytometry analyses. HEK293T cells are transfected with the same amount of reporter plasmid and SpCas9 in each comparison. The BFP sgRNA group consists of those transfected with reporter assay plasmid and a plasmid‐containing SpCas9 and BFP sgRNA. The control group contains those transfected with reporter assay plasmid and a plasmid‐containing SpCas9 and scaffold sgRNA. The reporter group is transfected with reporter assay plasmid and a neutral plasmid. Data show mean ± SD. *n* = 3 biological replicates. *P < 0.05, two‐tailed *t*‐tests. n.s., no significant difference.

In the NHEJ reporter assays, we inserted the BFP gRNA target with one or two additional base pairs to make GFP out of the reading frame (Fig. 5A). A proportion of the indels introduced upon NHEJ results in a frameshift that restores the functional GFP reading frame, thus probing the presence of NHEJ events in corresponding cells. However, because of the randomness of NHEJ repair, any design can hardly represent all possible NHEJ events [18, 24]. A series of distinct NHEJ reporter assays each indicating a different type of NHEJ repair outcome is needed to monitor all editing events. We generated NHEJ + 1 and NHEJ + 2, respectfully, with one or two base pair(s) frameshift, and NHEJ Stop + 1 with additional 3× stop codon (TAGTAGTAG; Fig. 5A). Among all the designs, the NHEJ + 1 assay reveals best performance with lowest background and highest efficiency when examining with the SpCas9 CRISPR system (Fig. 5B and Fig. S3). Notably, the NHEJ Stop + 1 design produces high background activity. This might be explained by that the premature stop codon ends translation from the beginning, which might lead to utilization of downstream start codons resulting in truncated proteins, still fluorescent, albeit at a much lower efficiency. We selected NHEJ + 1 assay for the following experiments.

The mTmG reporter assay was also tested in probing the NHEJ repair pathway. The design of the mTmG reporter assay involves the membrane localized Tomato (mT) and membrane localized GFP (mG). It was often used in conjunction with the Cre‐loxP recombinase system [46]. By replacing loxP, the Cre target sequence, with a gRNA target, Yang *et al*. [47] designed an mTmG assay compatible with the SpCas9 CRISPR system. Accordingly, we constructed an mTmG reporter using the BFP gRNA target to ensure the use for the three CRISPR‐Cas systems (Fig. S4). However, the mTmG assay as a double‐fluorescent system exhibits high background signal through flow cytometry analyses and was not selected for further analysis.

### Performance of reporter assays with three CRISPR‐Cas systems

**Fig. 6:**
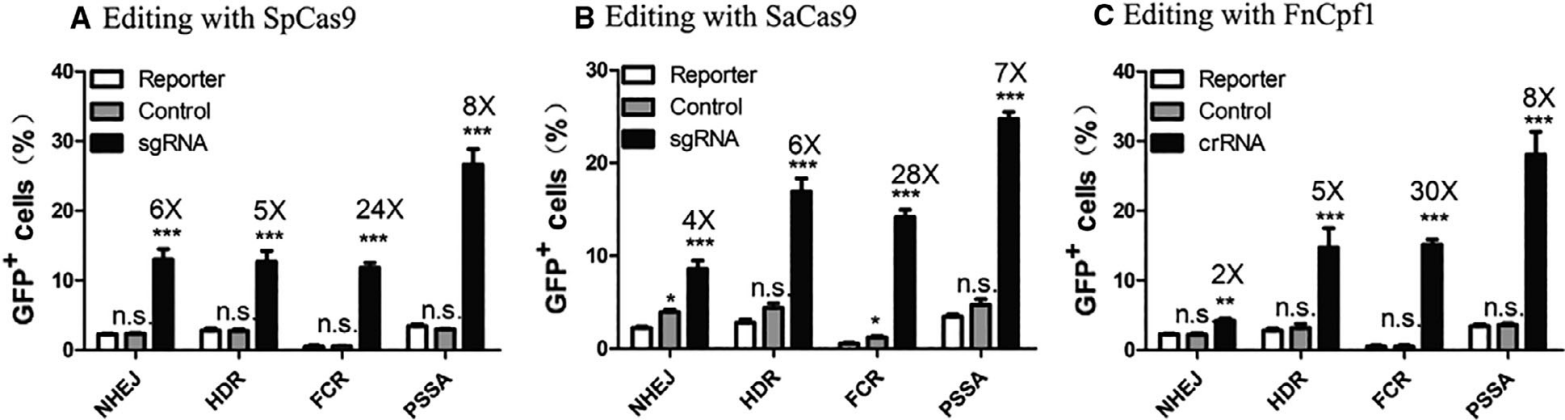
Analyses of various reporter assays in HEK293T cells. SpCas9 (A), SaCas9 (B) and FnCpf1 (C) are used to edit various GFP reporters, respectively. Included in these experiments are selected reporter assays with optimal performance from each category (refer to Figs S5–S7 for representative flow cytometry plots). GFP fluorescence indicates gene‐editing events. Efficiency of each reporter assay is quantified by counting postediting GFP‐positive cells from flow cytometry analyses. HEK293T cells are transfected with the same amount of reporter assay plasmid and Cas nuclease in each comparison. The sgRNA (crRNA) group is transfected with reporter assay plasmid and a plasmid containing corresponding Cas and sgRNA (crRNA). The control group consists of those transfected with reporter assay plasmid and a plasmid‐containing corresponding Cas and scaffold sgRNA (crRNA). The reporter group contains those transfected with reporter assay plasmid and a neutral plasmid. Data show mean ± SD. *n* = 3 biological replicates. *P < 0.05; **P < 0.01; ***P < 0.001, two‐tailed *t*‐tests. n.s., no significant difference.

After working on each of the aforementioned reporter assays individually, we compared the optimized designs of each reporter head‐to‐head in the same experiments (Fig. 6 and Figs S5–S7). All reporter assays produce statistically significant increase over background activity measured in GFP‐positive cell counts of 0.25–4%, regardless of which Cas is used (Fig. 6 and Figs S5–S7). Among the reporter assays, the pSSA assay shows the highest efficiency consistently across the three CRISPR‐Cas systems (Fig. 6). The result may be counterintuitive because NHEJ is the predominant DSB repair mechanism in mammalian cells [5]. However, this is consistent with the fact that an NHEJ assay can reflect only a proportion of editing events [18, 24].

**Fig. 7:**
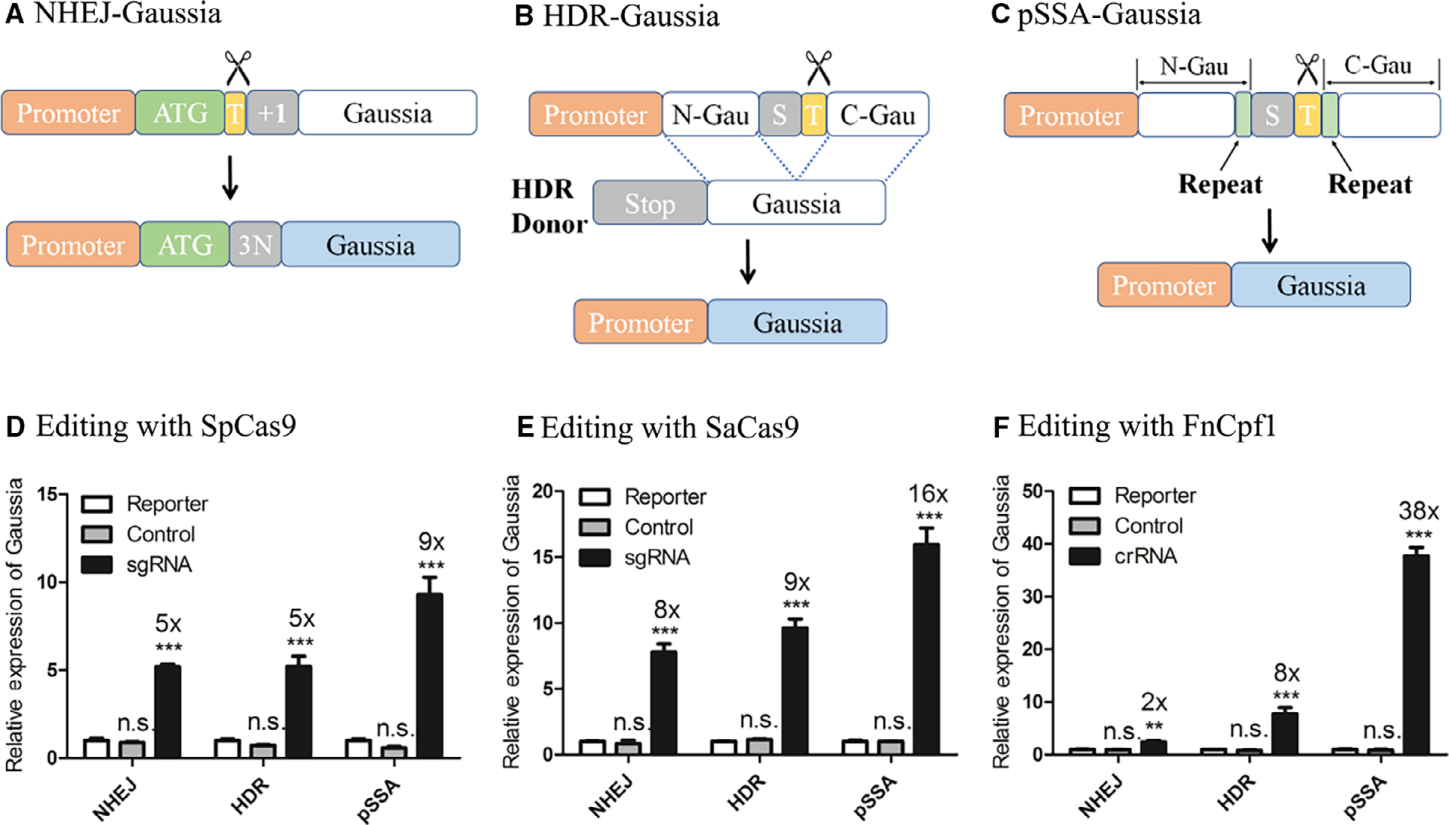
Design and examination of Gaussia reporter assays. Reporter assays with optimal performance are redesigned to adapt Gaussia luciferase (A–C). SpCas9 (D), SaCas9 (E) and FnCpf1 (F) are used to examine various Gaussia luciferase reporters, respectively. Gaussia expression indicates gene‐editing events. Efficiency of each reporter assay is quantified by measuring illuminance from Gaussia luciferase. HEK293T cells are transfected with the same amount of reporter assay plasmid and Cas nuclease in each comparison. The sgRNA (crRNA) group contains those transfected with reporter assay plasmid and a plasmid containing corresponding Cas and sgRNA (crRNA). The control group consists of those transfected with reporter assay plasmid and a plasmid containing corresponding Cas and scaffold sgRNA (crRNA). The reporter group is those transfected with reporter assay plasmid and a neutral plasmid. Data show mean ± SD. *n* = 3 biological replicates. **P < 0.01; ***P < 0.001, two‐tailed *t*‐tests. n.s., no significant difference.

Results from distinct CRISPR‐Cas systems are consistent for the HDR, FCR and pSSA assays. However, compared with the result with SpCas9 and SaCas9, the efficiency of the NHEJ assay is obviously low with the CRISPR‐FnCpf1 system (Figs 6 and 7). This can be explained by the distinct mechanisms of action of the three Cas. SpCas9 and SaCas9 intend to generate blunt ends at the DSB site that can be easily repaired through NHEJ [1, 2, 5]. FnCpf1, in contrast, produces overhangs that are less efficient for direct ligations in the NHEJ mechanism [4, 5, 9].

Although FCR reporter assay appears to have the lowest background activity and its fold of induction is the highest, an obvious caveat of FCR assay is the lack of flexibility for gRNA change. Therefore, we recommended the HDR reporter assay over FCR to probe the HDR mechanism when using customized gRNAs.

Taken together, good performances from the NHEJ, HDR and pSSA assays, each representing a distinct DNA repair mechanism, are achieved across three CRISPR‐Cas systems. The three reporter assays constitute an optimal toolbox, which are what we recommended for readers and what we chose for further analyses in this study.

### Gaussia reporter assays

A different reporter other than GFP is often needed because of the availability of analyzing instruments and the need for throughput. Performance of a good assay should not be altered by switching reporters. Luciferase reporters are among the most widely used, which can be analyzed with microplate readers and are amenable to high‐throughput applications [37, 48]. As a secreted luciferase derived from the copepod marine organism *Gaussia princeps*, Gaussia luciferase consists of a short coding sequence of 185 amino acids [49, 50, 51]. We replaced GFP with Gaussia luciferase in the NHEJ, HDR and pSSA reporter assays, respectively. In redesigning the NHEJ–Gaussia assay, we directly replaced the GFP reading frame with Gaussia luciferase reading frame (Fig. 7A), while more calculations guided with experience from the GFP reporter assays are needed for optimizing HDR and pSSA assays. In the HDR–Gaussia assay, the donor is the entire coding sequence of Gaussia luciferase, and the HDR template possesses a 369‐bp‐long N‐Gau and an 186‐bp‐long C‐Gau (Fig. 7B). In the pSSA‐Gaussia assay, N‐Gau and C‐Gau both consist of 386 bp, sharing a repeat of 184 bp (Fig. 7C). As a result, consistency is observed between the Gaussia reporter assays and those of GFP (Fig. 7D–F). Statistically significant increases over background of Gaussia expression are found with all three CRISPR‐Cas systems. The performance of Gaussia reporter assays can be further improved by switching to a more potent gRNA targeting adeno‐associated virus integration site 1 (AAVS1) locus, in which the BFP gRNA target site is replaced by an AAVS1 target (Fig. S8) [40, 41].

### Validation of reporter assays by chemical perturbations in DNA repair

**Fig. 8:**
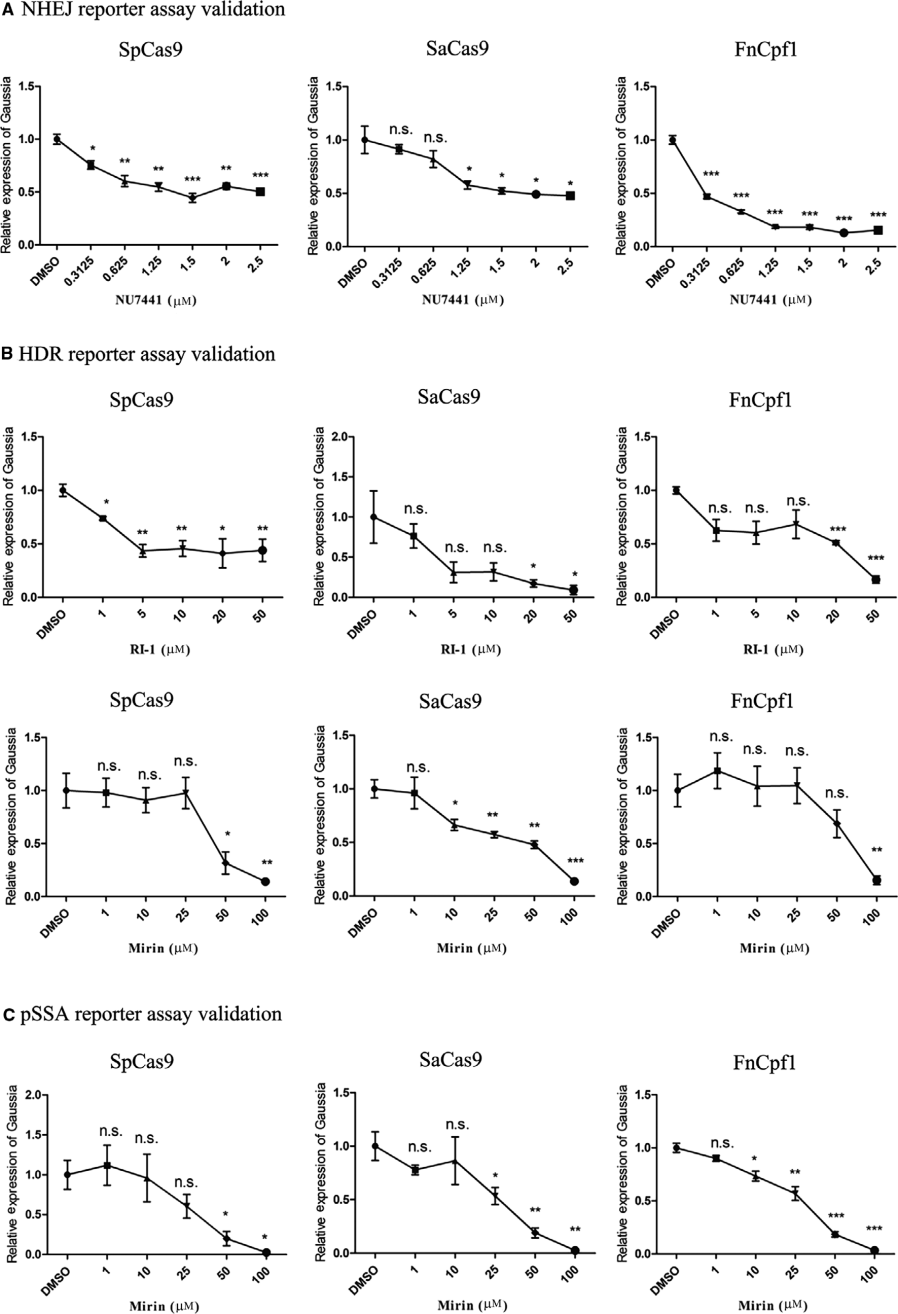
Validation of reporter assays by small molecules. (A) Validation of the NHEJ reporter assay by NU7441. (B) Validation of the HDR reporter assay by RI‐1 and Mirin. (C) Validation of the pSSA reporter assay by Mirin. HEK293T cells are transfected with each reporter assay, Cas and BFP targeted sgRNA (crRNA). NU7441, RI‐1 and Mirin of indicated concentrations are added 5 h after transfection. Forty‐eight hours afterward, gene‐editing events are qualified by measuring illuminance from Gaussia luciferase. DMSO serves as the vehicle. Gaussia readout is collected by microplate reader, and DMSO control data are normalized as 1. Data show mean ± SD. *n* = 3 biological replicates. *P < 0.05; **P < 0.01; ***P < 0.001, two‐tailed *t*‐tests. n.s., no significant difference.

We next used a series of small molecules, including NU7441, RI‐1 and Mirin, to validate the reporter assays. NU7441 was reported as a selective DNA‐PKcs inhibitor that antagonizes NHEJ activity [31, 33, 52, 53]. RI‐1 was reported to inhibit the function of the Rad51 protein and thus decrease HDR efficiency [35]. Mirin, as an inhibitor of MRN, was reported to decrease both HDR and SSA repair activities [34]. As shown in Fig. 8, inhibitory activities of NU7441 on NHEJ (Fig. 8A), RI‐1 on HDR (Fig. 8B), and Mirin on both HDR and SSA (Fig. 8B,C) are observed using Gaussia reporter assays before appearance of cell toxicity (Fig. S9). Results are consistent no matter which CRISPR‐Cas system is used to introduce DNA cleavages.

In addition to antagonizing NHEJ, NU7441 is expected to enhance HDR and SSA [31, 33, 52, 53]. Through the end resection process at the DSB site, the inhibition of DNA‐PKcs promotes the function of the MRN complex and the CtIP protein in creating resected DSB ends over blunt ends [16]. Because the NHEJ pathway repairs DSB with blunt ends, while other pathways, including HDR and SSA, take advantage of resected ends in mechanism, by inhibiting the function of DNA‐PK, HDR and SSA activities are predicted to be enhanced, while NHEJ activity is inhibited. Here we tested whether the reporter assays are capable of discriminating these different editing outcomes from NU7441 regulation. As shown in Fig. S10, activities from HDR, FCR and pSSA assays increase indeed. Consistency is observed between GFP and Gaussia reporters. Besides, apart from inhibiting HDR and SSA, Mirin also shows an enhancement in the NHEJ reporter assay (Fig. S11).

### Enrichment of cells with edited genome

**Fig. 9:**
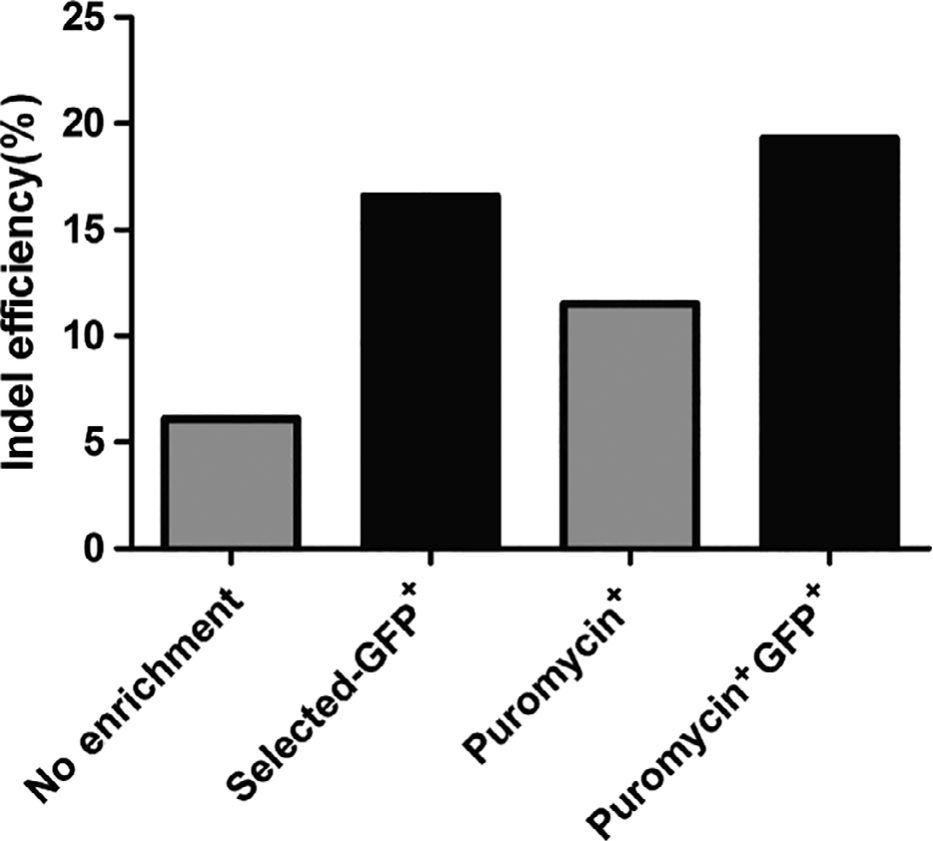
Enrichment of edited cells with pSSA‐GFP reporter. HEK293T/NLS‐Cas9 cell lines are transfected with gRNA targeting AAVS1 locus and a pSSA‐GFP reporter containing the AAVS1 target. Puromycin or DMSO vehicle control is added 48 h after transfection. GFP‐positive cells are collected using flow cytometry sorting. Indel efficiency is analyzed with TIDE. Sanger sequencing results from genomic DNA templates are shown in Fig. S12.

The CRISPR technology is widely used to generate cellular or organismal models via gene editing [54]. The CRISPR reporter assays can function as a surrogate for editing events, thus increasing efficiency and accuracy of gene editing [55]. Considering the excellent performance of the pSSA assay consistently in previous experiments, we next used the pSSA‐GFP assay to enrich cells that undergo the editing process. A HEK293T cell line stably expressing NLS‐SpCas9 was transfected with the pSSA‐GFP assay containing the gRNA target for the AAVS1 locus. GFP‐positive cells were sorted by flow cytometry 48 h after transfection. The ratio of AAVS1 editing events among GFP‐positive cells, according to the tracking of indels by decomposition (TIDE) analyses [38], are two to four times higher than that of unsorted cells (Fig. 9 and Fig. S12). Moreover, the gRNA plasmid used in this experiment also expresses puromycin‐resistant gene, and we also enriched transfected cells with puromycin selection. By comparing puromycin‐resistant and GFP‐positive cells, enrichment using the pSSA‐GFP reporter assay has shown better efficiency. Lastly, a further improvement of enrichment efficiency can be achieved when combining puromycin selection and GFP sorting (Fig. 9).

## Discussion

In this study, we have developed a series of reporter assays for distinct gene‐editing mechanisms, including NHEJ, HDR and SSA. The outcome and efficiency of editing can be intuitively detected through GFP or Gaussia expression. Taking advantage of the three CRISPR‐Cas systems and their differences in mechanism of generating DSB, this reporter assay toolbox can study DSB repair from both blunt ends and resected ends. They are flexible in the ability to adopt different targets, reporter genes and Cas. Because the gRNA target site is independent of the reporter gene sequence in our designs, it can be easily changed to serve the need of future research in studying other genomic target sites. Moreover, with additional PAM being introduced at the corresponding region, the reporter assays can be further optimized and will no longer be limited to the three CRISPR‐Cas systems aforementioned.

We started with the same GFP reporter gene, the same BFP gRNA and the same flow cytometry measurements and comparatively examined different reporter assays in the same experiments, while the molar amounts of Cas, gRNA, and reporter assay constructs were matched across all samples. NU7441, RI‐1 and Mirin, reported as inhibitors for distinct DSB repair mechanisms [33, 34, 35, 52], were used to validate the capability of the NHEJ, HDR and pSSA assays as probes for corresponding DNA repair mechanisms. These GFP reporter assays produced consistent results. The controlled experiments allow us to compare different assays in an unbiased manner. Overall, the optimized pSSA reporter assay displays best performances consistently throughout this study. Therefore, we used it to enrich cells with an edited AAVS1 locus. Because the CRISPR reporter assays can function as a surrogate for editing events, the use of reporter assays in cell enrichment can increase the efficiency and accuracy of gene editing [55], and such a method is proved to be effective according to our results.

Moreover, although we did not follow up on FCR assay because of its limited flexibility in choosing gRNAs in this report, its great sensitivity helped us while developing drug‐inducible CRISPR systems we named “HIT” [40, 41]. The FCR assay can play a role as a sensitive and robust assay for further development of more CRISPR editing tools when a gRNA target can be found in close proximity of the amino acid substitution site in BFP.

Notably, Gaussia reporter assays appear to be more sensitive than GFP reporter assays, as indicated by often higher folds of induction upon DNA cleavage (Figs 6 and 7). This probably reflects the distinct manners of these two reporters in producing signals and the ways we measure them. Chemical luminescence signal from Gaussia luciferase is yielded through an enzymatic process wherein amplification occurs, which fluorescent signal from GFP lacks. Moreover, Gaussia signal is measured at the molecular level, and GFP signal is quantified at the cellular level as positive cell percentiles. The higher sensitivity of Gaussia reporters and its compatibility with high‐throughput format favors their applications over GFP reporters in many situations.

The reporter assays in this study can be further developed to serve more sophisticated needs. For example, orthogonal fluorescent protein or luciferase reporters can be multiplexed to probe distinct DSB repair mechanisms simultaneously [17, 18, 19, 22, 23, 24, 25, 27, 28, 29].

Transient transfection was used throughout this study to deliver the reporter constructs. Unlike stable genome integration of reporter constructs, in which the generation of cell line has to go through a laborious process, transient transfection provides the possibility for massive tests in a short term. It is worth mentioning that the copy number of reporter constructs in transiently transfected cells is normally much higher than those in cells selected through stable genome integration. Consequently, background activity is higher upon transient transfection. This exists as a common challenge for reporter assays. Only those with good signal‐to‐noise ratio can be used via transient transfection. Otherwise, a tedious stable cell line generation has to be carried out.

In this study, we carried out validation and fluorescent enrichment experiments for cellular editing. These reporter assays can find broader utility beyond. For example, the capability of these reporter assays, when combined, to distinguish distinct DNA repair pathways would provide value in mechanistic studies. The compatibility of these reporter assays with high‐throughput use would allow large‐scale screening of new genes and new drugs regulating CRISPR systems and DNA repair. Such efforts will potentially translate into better understanding of CRISPR systems and DNA repair mechanisms, improvement in genome‐editing efficiency for both research and clinical purposes, and opportunities for cancer therapies targeting compromised DNA repair activity [28, 32, 56, 57, 58].

## Author contributions

YW and BZ conceived and supervised this study. LC designed experiments. LC and HG performed experiments. LC analyzed data. All authors wrote and revised the manuscript.

## Conflict of interest

The authors declare no conflict of interest.

## Supporting information


**Fig. S1**. Representative flow cytometry plots using SpCas9, related to Fig. 3B.
**Fig. S2**. Representative flow cytometry plots using SpCas9, related to Fig. 4B.
**Fig. S3**. Representative flow cytometry plots using SpCas9, related to Fig. 5B.
**Fig. S4**. mTmG reporter assay. CRISPR cleavages in the target region (shown in yellow) are symbolized by the scissors. The CRISPR‐mediated excision of the membrane‐targeted tandem dimer Tomato (mT, shown in red) sequence and the stop sequence (PolyA, shown in gray) allows the expression of membrane‐targeted GFP (mG, shown in green). Results are obtained using SpCas9, SaCas9 and FnCpf1. GFP fluorescence indicates gene‐editing events. Efficiency of each reporter assay is quantified by counting postediting GFP‐positive cells from flow cytometry analyses. HEK293T cells are transfected with same amount of reporter assay plasmid and Cas nuclease in each comparison. sgRNA (crRNA) group is transfected with reporter assay plasmid and a plasmid containing corresponding Cas and sgRNA (crRNA). Control group contains those transfected with reporter assay plasmid and a plasmid containing corresponding Cas and scaffold sgRNA (crRNA). The reporter group consisted of transfection with reporter assay plasmid and a neutral plasmid. Data show mean ± SD. *n* = 3 biological replicates. *P < 0.05; **P < 0.01; ***P < 0.001, two‐tailed *t*‐tests. n.s., no significant difference.
**Fig. S5**. Representative flow cytometry plots using SpCas9, related to Fig. 6A.
**Fig. S6**. Representative flow cytometry plots using SaCas9, related to Fig. 6B.
**Fig. S7**. Representative flow cytometry plots using FnCpf1, related to Fig. 6C.
**Fig. S8**. Comparison of reporter assays using different gRNA targets. (A) Results from the NHEJ assay. (B) Results from the HDR assay. (C) Results from the pSSA assay. Efficiency of each reporter assay containing BFP or AAVS1 target is quantified by measuring illuminance from Gaussia luciferase. HEK293T cells are transfected with the same amount of reporter assay plasmid and SpCas9 in each comparison. The sgRNA group contains those transfected with reporter assay plasmid and a plasmid containing SpCas9 and BFP/AAVS1 sgRNA. The control group is transfected with reporter assay plasmid and a plasmid containing SpCas9 and scaffold sgRNA. The reporter group consists of those transfected with reporter assay plasmid and a neutral plasmid. Data show mean ± SD. *n* = 3 biological replicates. *P < 0.05; **P < 0.01; ***P < 0.001, two‐tailed *t*‐tests. (D) Comparison of BFP and AAVS1 sgRNA efficiency (results from TIDE analyses). HEK293T cells with transgenic BFP stably integrated are transfected with a plasmid containing SpCas9 and BFP/AAVS1 sgRNA to compare the efficiency of sgRNAs. Puromycin is added 48 h after transfection to enrich successfully transfected cells. Genomic DNA templates are obtained by cell lysis and PCR amplified. Indel efficiency is analyzed with TIDE and shown as “total eff.” Sanger sequencing results from genomic DNA templates are also shown. Expected cutting sites are labeled with red arrows. n.s., no significant difference.
**Fig. S9**. Cell viability under the treatment of NU7441, RI‐1 and Mirin. HEK293T cells are seeded with the same amount in each well of a 96‐well dish and are cultured in indicated concentrations of NU7441 or Mirin or RI‐1. Cell numbers are consistent in each experiment. Forty‐eight hours after treatment, 10 μL cell counting solution is added in each well and incubated for 1 h at 37°C. The absorbance at 450 nm is determined with a plate reader. The cell viability of wells containing DMSO‐treated cells and wells containing medium only are set as 100% and 0%, respectively; all other viabilities are normalized to these values. Data show mean ± SD. *n* = 3 biological replicates. *P < 0.05; **P < 0.01; ***P < 0.001, two‐tailed *t*‐tests. n.s., no significant difference.
**Fig. S10**. NU7441 performance on HDR and SSA. HEK293T cells are transfected with each reporter, SpCas9, and BFP targeted sgRNA. NU7441 of indicated concentrations are added 5 h after transfection. Forty‐eight hours afterward, gene‐editing events are qualified by counting the GFP‐positive cells (A, B, and D) through flow cytometry analyses or measuring illuminance from Gaussia luciferase (C and E). DMSO serves as the vehicle. Gaussia readout is collected by micro‐plate reader, and DMSO data are normalized as 1. Data show mean ± SD. *n* = 3 biological replicates. *P < 0.05; **P < 0.01; ***P < 0.001, two‐tailed *t*‐tests. n.s., no significant difference.
**Fig. S11**. Mirin performance on NHEJ repair. HEK293T cells are transfected with NHEJ reporter assay, Cas, and BFP targeted sgRNA (crRNA). Mirin of indicated concentrations is added 5 h after transfection. Forty‐eight hours afterward, gene‐editing events are qualified by measuring illuminance from Gaussia luciferase. DMSO serves as the vehicle. Gaussia readout is collected by microplate reader, and DMSO control data are normalized as 1. Data show mean ± SD. *n* = 3 biological replicates. *P < 0.05; **P < 0.01; ***P < 0.001, two‐tailed *t*‐tests. n.s., no significant difference.
**Fig. S12**. Sanger sequencing results from genomic DNA templates, related to Fig. 9. Expected cleavage sites are labeled with red arrows. HEK293T/NLS‐Cas9 cell lines are transfected with gRNA targeting the *AAVS1* locus and a pSSA‐GFP reporter containing the AAVS1 gRNA target. Puromycin or DMSO vehicle control is added 48 h after transfection. GFP‐positive cells are collected using flow cytometry sorting.
**Table S1**. Summary of gene‐editing reporter assays.
**Table S2**. Primers used in this study.
**Appendix S1**. Supplementary DNA sequences.
**Appendix S2**. Supplementary sequences of amino acids.

## Data Availability

The data that support the findings of this study are available in the figures and the Supporting Information of this article.
