# Supplementary material for: Comprehensive optimization of a reporter assay toolbox for three distinct CRISPR‐Cas systems
Source: FEBS Open Bio. 2021 Jun 9;11(7):1965–80. doi: 10.1002/2211-5463.13198 (PMC8255852; doi:10.1002/2211-5463.13198)
Supplement: Supplementary file 1 — Fig. S1. Representative flow cytometry plots using SpCas9, related to Fig. 3B. Fig. S2. Representative flow cytometry plots using SpCas9, related to Fig. 4B. Fig. S3. Representative flow cytometry plots using SpCas9, related to Fig. 5B. Fig. S4. mTmG reporter assay. CRISPR cleavages in the target region (shown in yellow) are symbolized by the scissors. The CRISPR‐mediated excision of the membrane‐targeted tandem dimer Tomato (mT, shown in red) sequence and the stop sequence (PolyA, shown in gray) allows the expression of membrane‐targeted GFP (mG, shown in green). Results are obtained using SpCas9, SaCas9 and FnCpf1. GFP fluorescence indicates gene‐editing events. Efficiency of each reporter assay is quantified by counting postediting GFP‐positive cells from flow cytometry analyses. HEK293T cells are transfected with same amount of reporter assay plasmid and Cas nuclease in each comparison. sgRNA (crRNA) group is transfected with reporter assay plasmid and a plasmid containing corresponding Cas and sgRNA (crRNA). Control group contains those transfected with reporter assay plasmid and a plasmid containing corresponding Cas and scaffold sgRNA (crRNA). The reporter group consisted of transfection with reporter assay plasmid and a neutral plasmid. Data show mean ± SD. n = 3 biological replicates. *P < 0.05; **P < 0.01; ***P < 0.001, two‐tailed t‐tests. n.s., no significant difference. Fig. S5. Representative flow cytometry plots using SpCas9, related to Fig. 6A. Fig. S6. Representative flow cytometry plots using SaCas9, related to Fig. 6B. Fig. S7. Representative flow cytometry plots using FnCpf1, related to Fig. 6C. Fig. S8. Comparison of reporter assays using different gRNA targets. (A) Results from the NHEJ assay. (B) Results from the HDR assay. (C) Results from the pSSA assay. Efficiency of each reporter assay containing BFP or AAVS1 target is quantified by measuring illuminance from Gaussia luciferase. HEK293T cells are transfected with the same amount of reporter [file FEB4-11-1965-s001.doc]

**Comprehensive optimization of reporter assay toolbox for three distinct CRISPR-Cas systems**

Li Chen^1,2^, Haoyuan Gao^3,4^, Bing Zhou^1,2,5*^, Yu Wang^3*^

1. State Key Laboratory of Stem Cell and Reproductive Biology, Institute of Zoology, Chinese Academy of Sciences, Beijing, China.

2. University of Chinese Academy of Sciences, Beijing, China.

3. College of Life Sciences and Oceanography, Shenzhen University, Shenzhen, China.

4. Department of Biology, Oberlin College, Oberlin, OH 44074, USA.

5. Institute for Stem Cell and Regeneration, Chinese Academy of Sciences, Beijing, China.

*. Corresponding Authors: Yu Wang: College of Life Sciences and Oceanography, Shenzhen University, 1066 Xueyuan Avenue, Nanshan District, Shenzhen, China. Phone: +86-18612208166; E-mail: [yuwang@post.harvard.edu](mailto:yuwang@post.harvard.edu); or Bing Zhou: Institute of Zoology, Chinese Academy of Sciences, 1 Beichen West Road, Beijing 100101, China. E-mail: [zhoubing@ioz.ac.cn](http://sourcedb.ioz.cas.cn/zw/zjrc/201808/t20180810_5054675.html).

Supporting Information:

Supplementary Figure S1-S12

Note 1. Supplementary DNA sequences

Note 2. Supplementary sequences of amino acids

Table S1. Summary of gene editing reporter assays

Table S2. Primers used in this study

**Supplementary Figures**


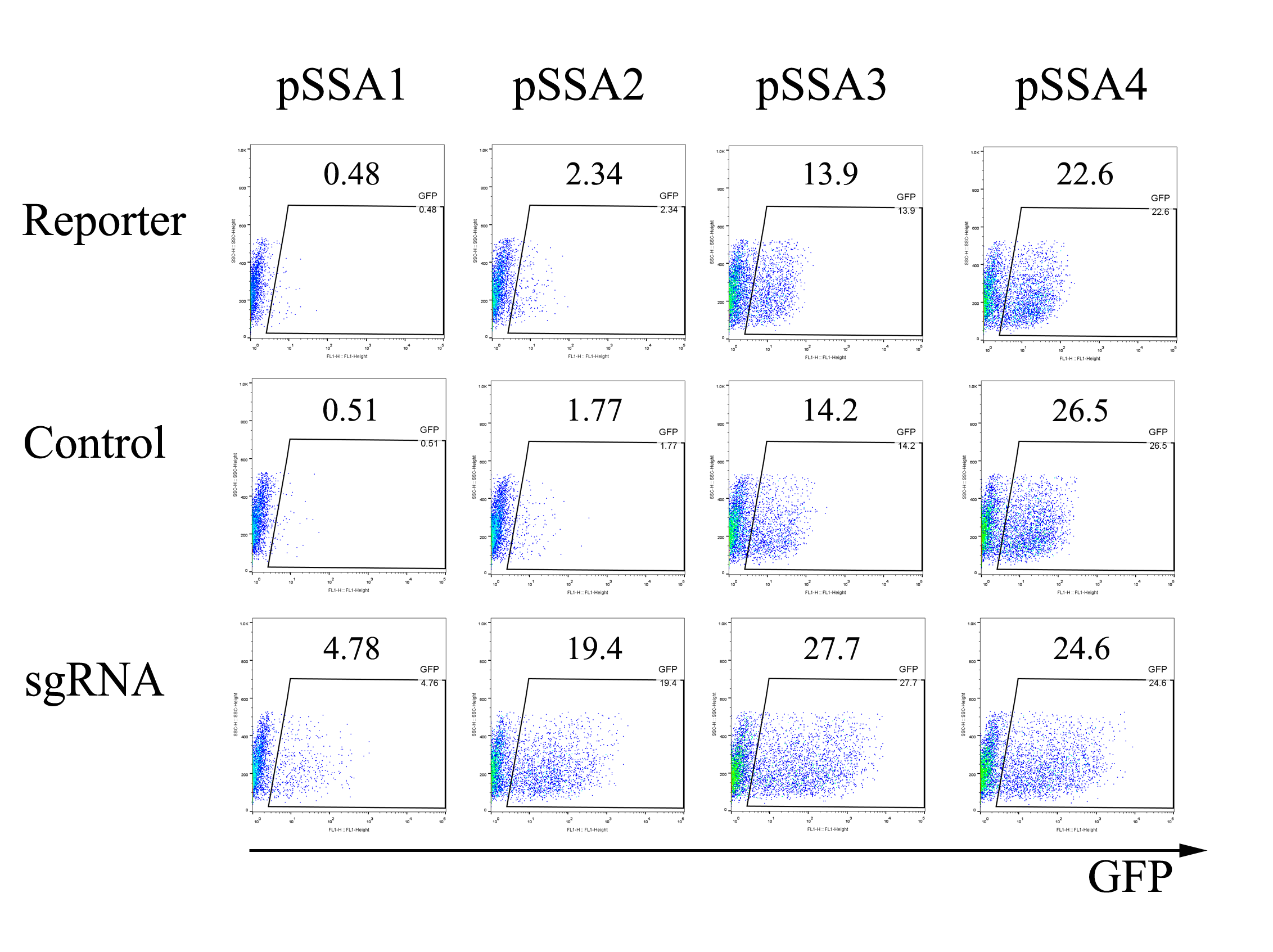


**Figure S1. Representative flow cytometry plots using SpCas9, related to Figure 3B.**


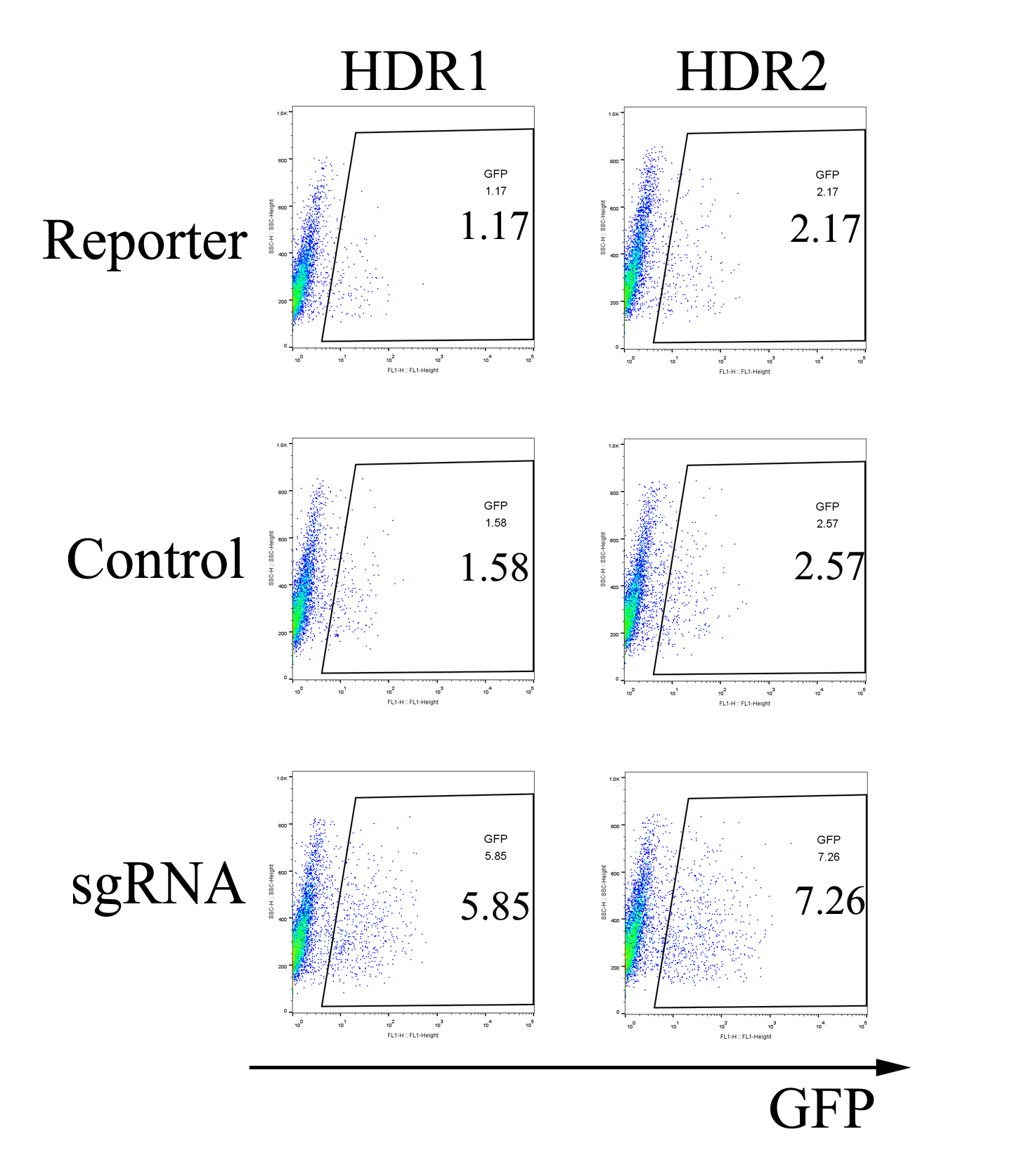


**Figure S2. Representative flow cytometry plots using SpCas9, related to Figure 4B.**


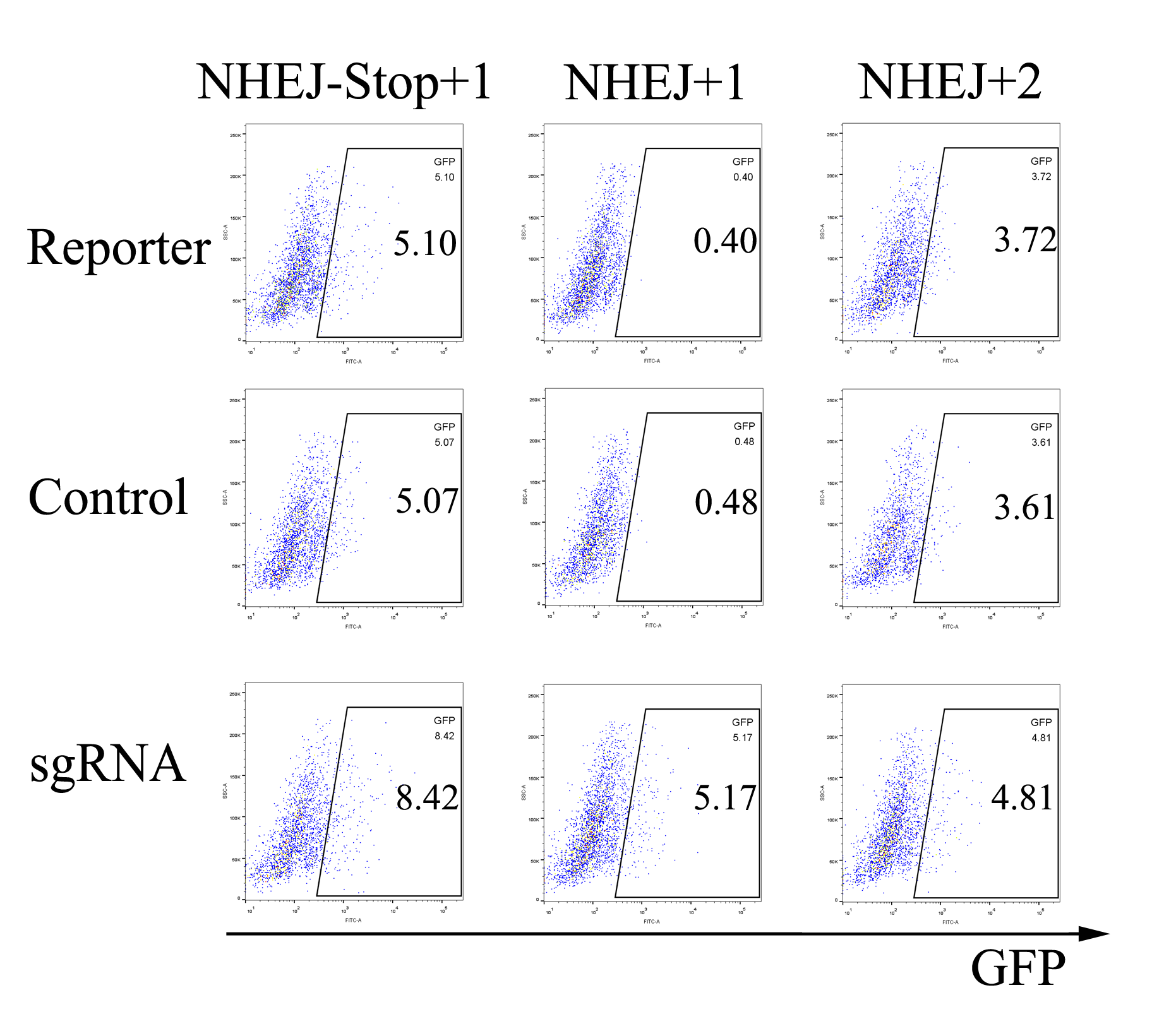


**Figure S3. Representative flow cytometry plots using SpCas9, related to Figure 5B.**


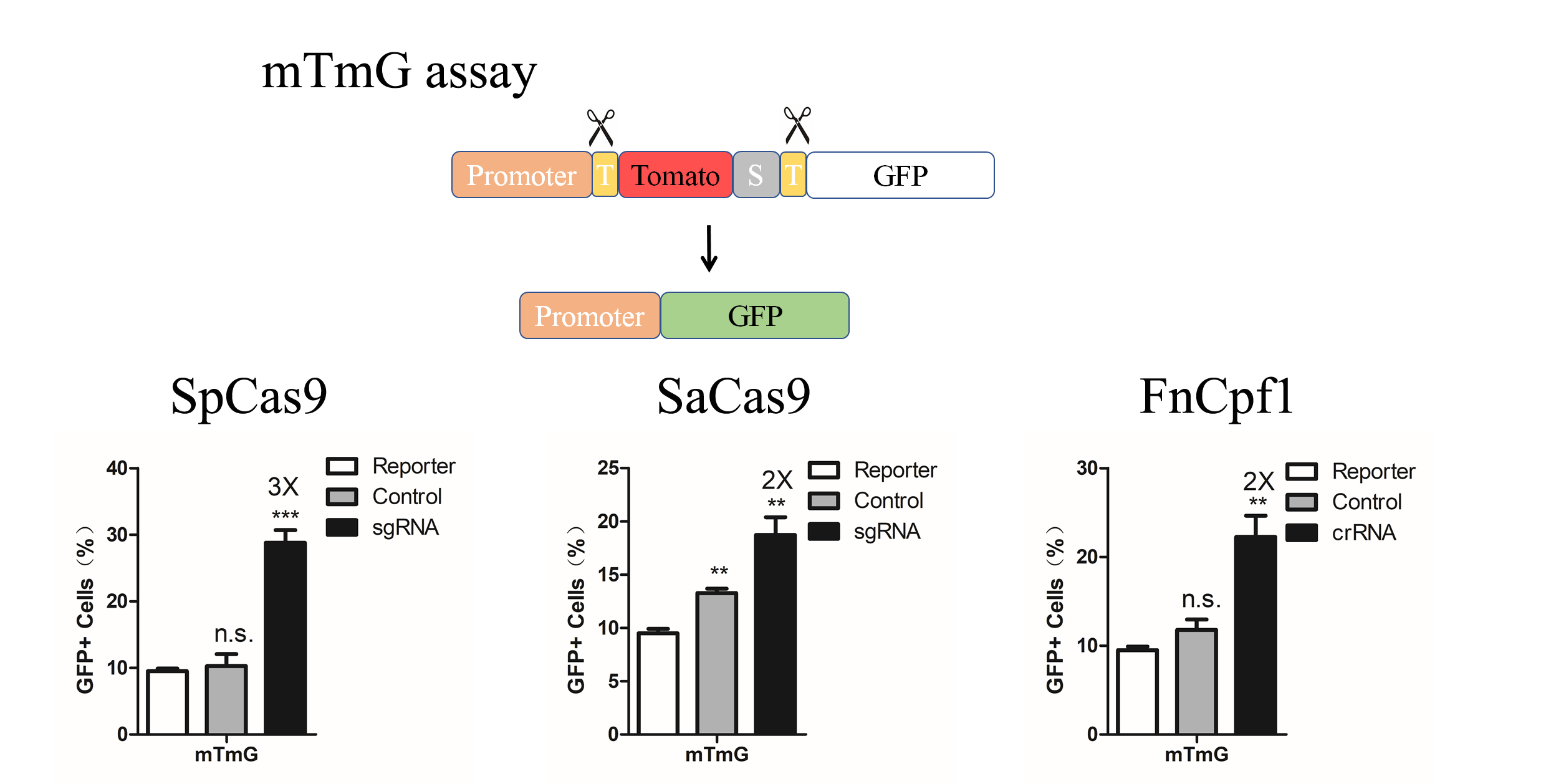


**Figure S4. mTmG reporter assay.** CRISPR cleavages in the target region (shown in yellow) are symbolized by the scissor. The CRISPR mediated excision of the membrane-targeted tandem dimer Tomato (mT, shown in red) sequence and the stop sequence (PolyA, shown in gray) allows the expression of membrane-targeted GFP (mG, shown in green). Results are obtained using SpCas9, SaCas9, and FnCpf1. GFP fluorescence indicates gene editing events. Efficiency of each reporter assay is quantified by counting post-editing GFP-positive cells from flow cytometry analyses. HEK293T cells are transfected with same amount of reporter assay plasmid and Cas nuclease in each comparison. sgRNA (crRNA) group are transfected with reporter assay plasmid and a plasmid containing corresponding Cas and sgRNA (crRNA). Control group contain those transfected with reporter assay plasmid and a plasmid containing corresponding Cas and scaffold sgRNA (crRNA). Reporter group consist of transfected with reporter assay plasmid and a neutral plasmid. Data shows mean ± SD. n = 3 biological replicates. n.s.: no significant difference; *P < 0.05; **P < 0.01; ***P < 0.001; two tailed t-tests.


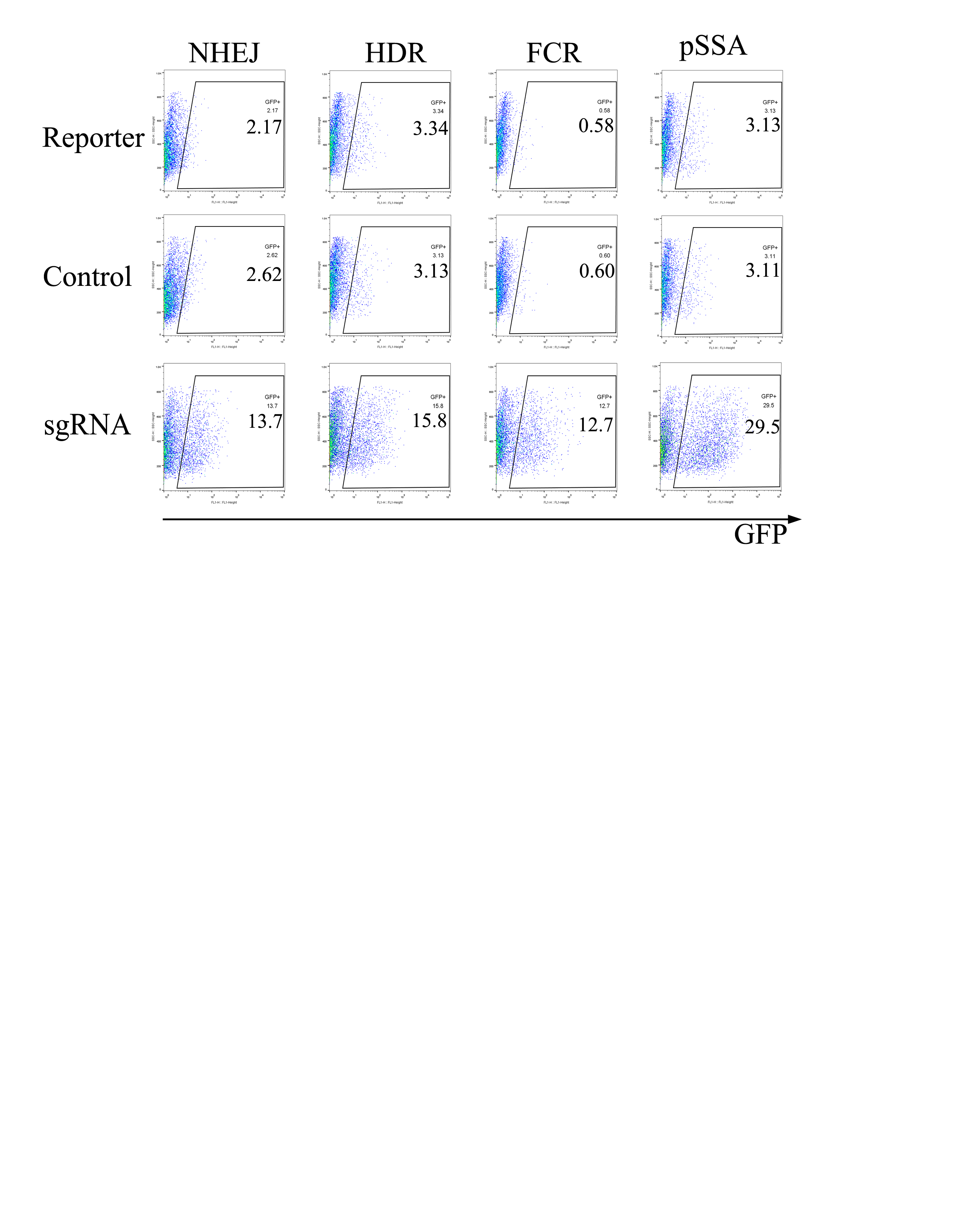


**Figure S5. Representative flow cytometry plots using SpCas9, related to Figure 6A.**


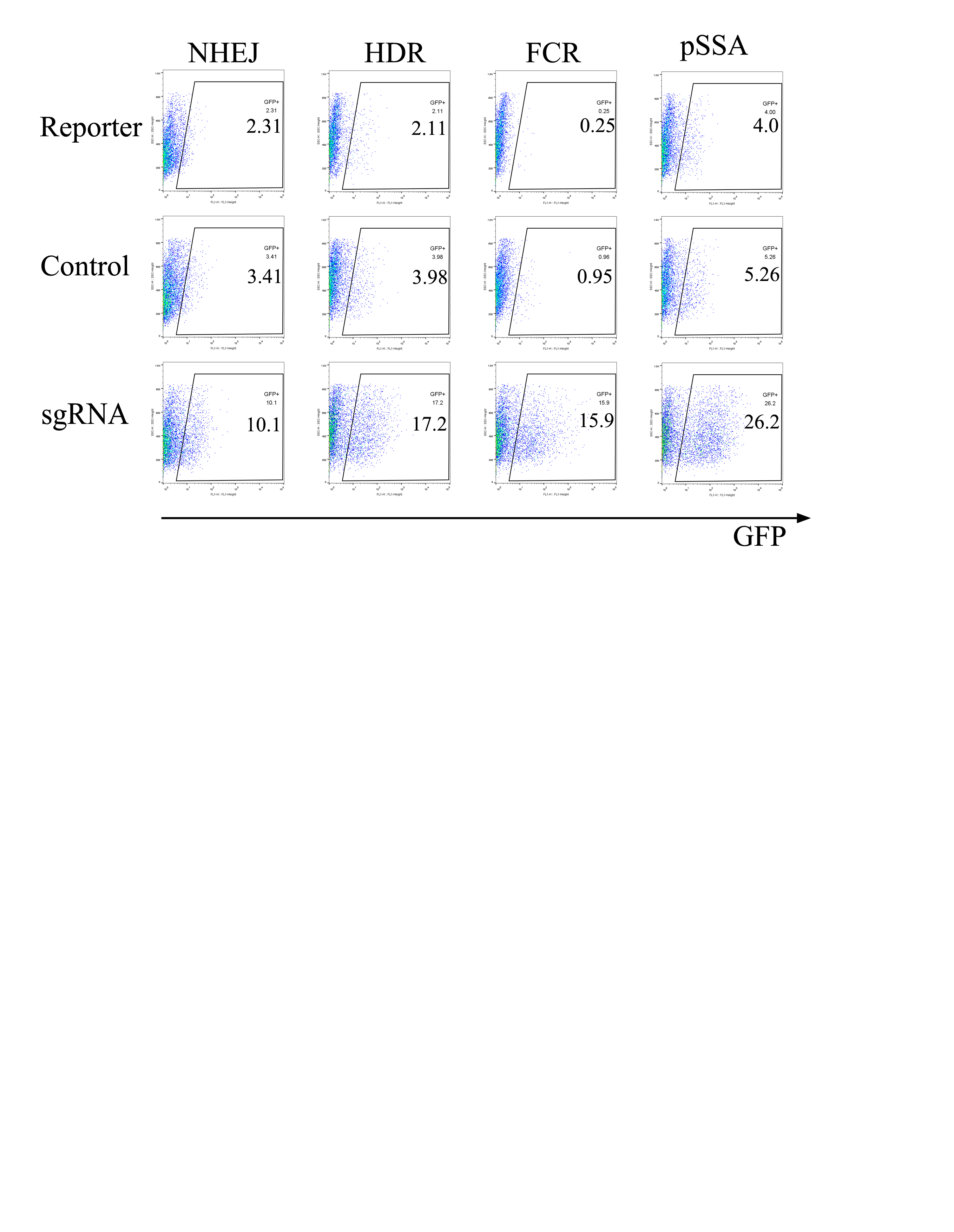


**Figure S6. Representative flow cytometry plots using SaCas9, related to Figure 6B.**


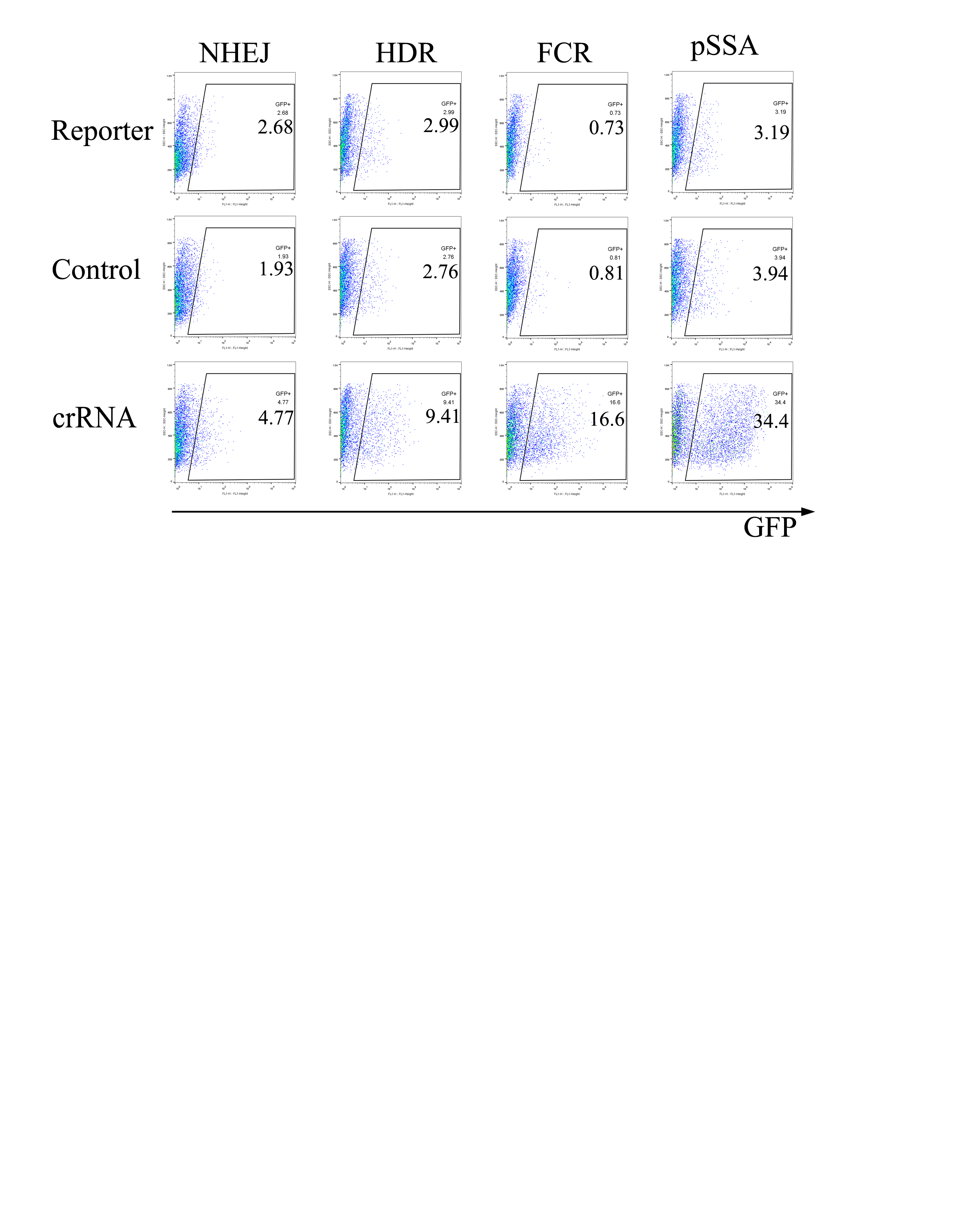


**Figure S7. Representative flow cytometry plots using FnCpf1, related to Figure 6C.**


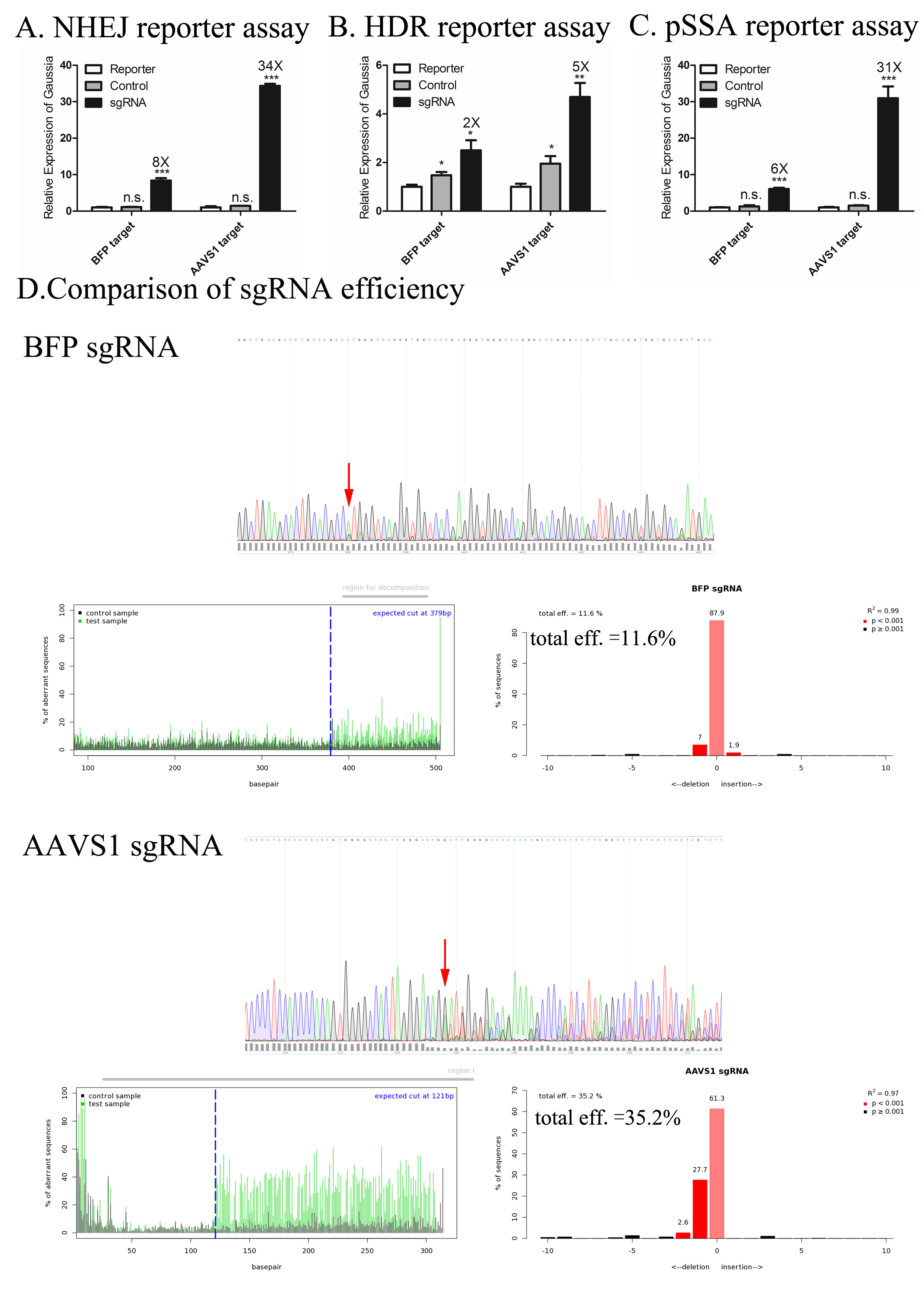


**Figure S8. Comparison of reporter assays using different gRNA targets.** **A.** Results from the NHEJ assay. **B.** Results from the HDR assay. **C.** Results from the pSSA assay. Efficiency of each reporter assay containing BFP or AAVS1 target is quantified by measuring illuminance from Gaussia luciferase. HEK293T cells are transfected with same amount of reporter assay plasmid and SpCas9 in each comparison. sgRNA group contain those transfected with reporter assay plasmid and a plasmid containing SpCas9 and BFP/AAVS1 sgRNA. Control group are transfected with reporter assay plasmid and a plasmid containing SpCas9 and scaffold sgRNA. Reporter group consist of those transfected with reporter assay plasmid and a neutral plasmid. Data shows mean ± SD. n = 3 biological replicates. n.s.: no significant difference; *P < 0.05; **P < 0.01; ***P < 0.001; two tailed t-tests. **D.** Comparison of BFP and AAVS1 sgRNA efficiency (results from TIDE analyses). HEK293T cells with transgenic BFP stably integrated are transfected with a plasmid containing SpCas9 and BFP/AAVS1 sgRNA to compare the efficiency of sgRNAs. Puromycin is added 48 hours after transfection to enrich successfully transfected cells. Genomic DNA templates are obtained by cell lysis and PCR amplified. Indel efficiency is analyzed with TIDE and shown as “total eff.” Sanger sequencing results from genomic DNA templates are also shown. Expected cutting sites are labeled with red arrows.


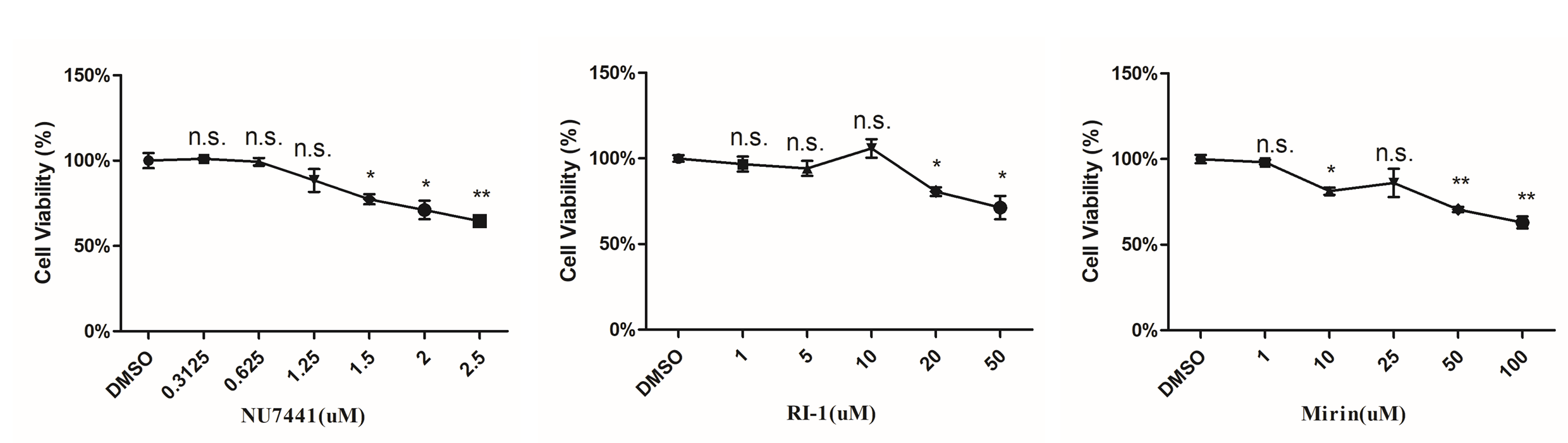


**Figure S9. Cell viability under the treatment of NU7441, RI-1, and Mirin.** HEK293T cells are seeded with same amount in each well of 96-well dish and are cultured in indicated concentrations of NU7441 or Mirin or RI-1. Cell numbers are consistent in each experiment. 48 hours after treatment, 10ul cell counting solution are added in each well and incubated 1 hour at 37℃. The absorbance at 450 nm is determined with a plate reader. The cell viability of wells containing DMSO treated cells and wells containing medium only are set as 100% and 0%, respectively, all other viabilities are normalized to these values. Data shows mean ± SD. n = 3 biological replicates. n.s.: no significant difference; *P < 0.05; **P < 0.01; ***P < 0.001; two tailed t-tests.


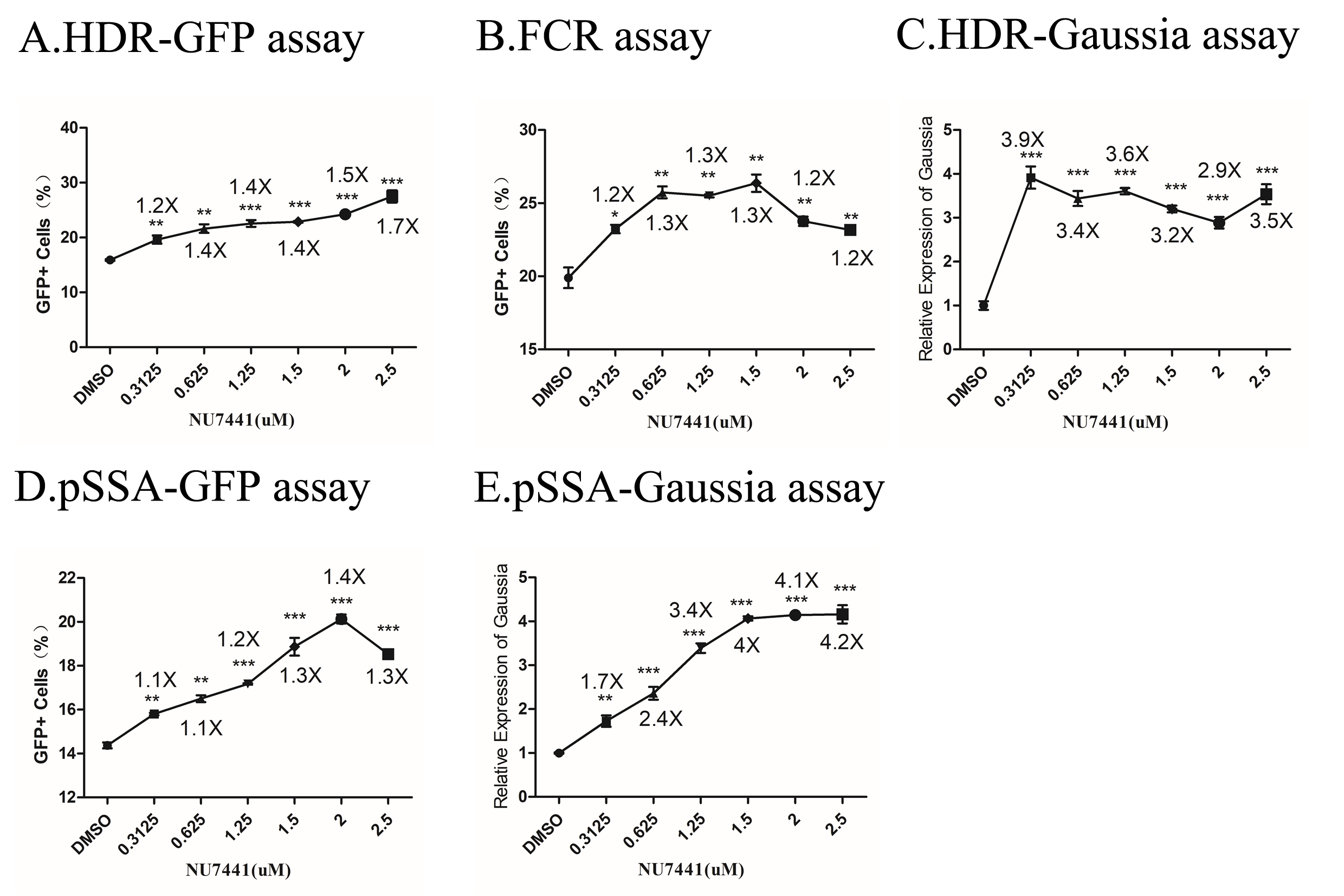


**Figure S10. NU7441 performance on HDR and SSA.** HEK293T cells are transfected with each reporter, SpCas9, and BFP targeted sgRNA. NU7441 of indicated concentrations are added 5 hours after transfection. 48 hours afterward, gene editing events are qualified by counting the GFP positive cells (**A, B, and D**) through flow cytometry analyses or measuring illuminance from Gaussia luciferase (**C and E**). DMSO serves as the vehicle. Gaussia readout is collected by micro-plate reader and DMSO data is normalized as 1. Data shows mean ± SD. n = 3 biological replicates. n.s.: no significant difference; *P < 0.05; **P < 0.01; ***P < 0.001; two tailed t-tests.


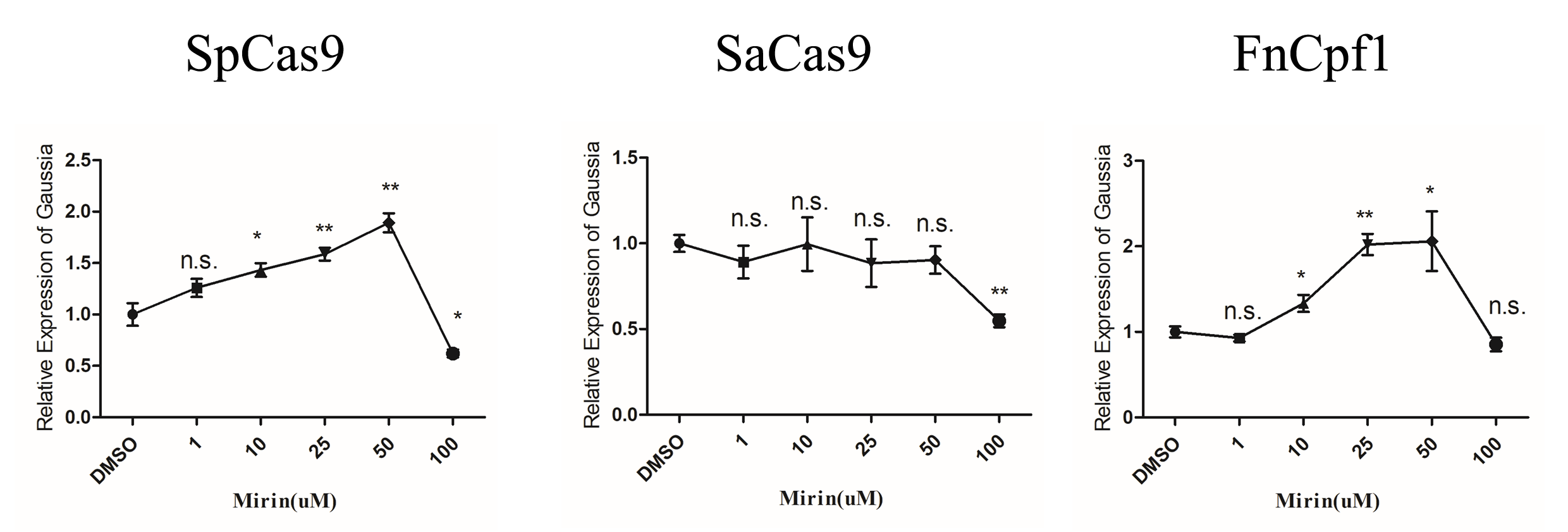


**Figure S11. Mirin performance on NHEJ repair.** HEK293T cells are transfected with NHEJ reporter assay, Cas, and BFP targeted sgRNA (crRNA). Mirin of indicated concentrations are added 5 hours after transfection. 48 hours afterward, gene editing events are qualified by measuring illuminance from Gaussia luciferase. DMSO serves as the vehicle. Gaussia readout is collected by micro-plate reader and DMSO control data is normalized as 1. Data shows mean ± SD. n = 3 biological replicates. n.s.: no significant difference; *P < 0.05; **P < 0.01; ***P < 0.001; two tailed t-tests.


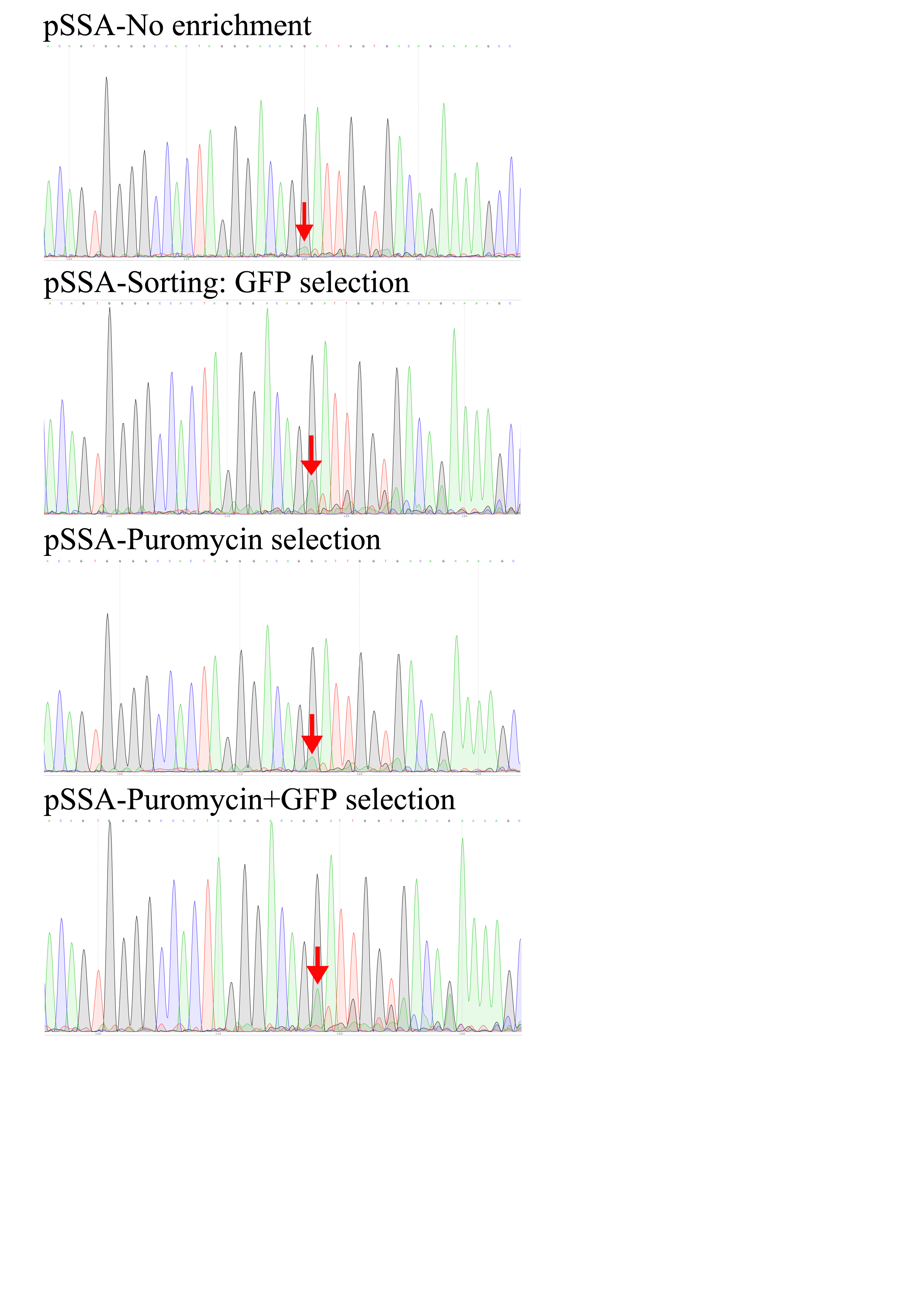


**Figure S12. Sanger sequencing results from genomic DNA templates, related to Figure 9.** Expected cleavage sites are labeled with red arrows. HEK293T/NLS-Cas9 cell lines are transfected with gRNA targeting the *AAVS1* locus and a pSSA-GFP reporter containing the AAVS1 gRNA target. Puromycin or DMSO vehicle control is added 48 hours after transfection. GFP-positive cells are collected using flow cytometry sorting.

**Note 1. Supplementary DNA sequences**

All sequences are in the 5’ to 3’ direction. BFP targets are highlighted in yellow.

>BFP open reading frame in FR reporter plasmid

atggtgagcaagggcgaggagctgttcaccggggtggtgcccatcctggtcgagctggacggcgacgtaaacggccacaagttcagcgtgtccggcgagggcgagggcgatgccacctacggcaagctgaccctgaagttcatctgcaccaccggcaagctgcccgtgccctggcccactttagtgaccaccctgacccatggcgtgcagtgcttcagccgctaccccgaccacatgaagcagcacgacttcttcaagtccgccatgcccgaaggctacgtccaggagcgcaccatcttcttcaaggacgacggcaactacaagacccgcgccgaggtgaagttcgagggcgacaccctggtgaaccgcatcgagctgaagggcatcgacttcaaggaggacggcaacatcctggggcacaagctggagtacaactacaacagccacaacgtctatatcatggccgacaagcagaagaacggcatcaaggtgaacttcaagatccgccacaacatcgaggacggcagcgtgcagctcgccgaccactaccagcagaacacccccatcggcgacggccccgtgctgctgcccgacaaccactacctgagcacccagtccaagctgagcaaagaccccaacgagaagcgcgatcacatggtcctgctggagttcgtgaccgccgccgggatcactctcggcatggacgagctgtacaagtga

(Mutant is highlighted in red.)

>FCR donor

gccacctacggcaagctgaccctgaagttcatctgcaccaccggcaagctgcccgtgccctggcccaccctcgtgaccaccctgacgtacggcgtgcagtgcttcagccgctaccccgaccacatga

>pSSA1-GFP reading frame

atggtgagcaagggcgaggagctgttcaccggggtggtgcccatcctggtcgagctggacggcgacgtaaacggccacaagttcagcgtgtccggcgagggcgagggcgatgccacctacggcaagctgaccctgaagttcatctgcaccaccggcaagctgcccgtgccctggcccaccctcgtgaccaccctgacctacggcgtgcagtgcttcagccgctaccccgaccacatgaagcagcacgacttcttcaagtccgccatgcccgaaggctacgtccaggagcgcaccatcttctgataaggatcccgggatcctaagcttgatgcatagcttgagtattctaacgcgtcacctaaatagcttggcgtaatcatggtcatagctgtttcctgtgtgaaattgttatccgctcacaattccacacaacatacgagccggaagcataaagtgtaaagcctggggtgcctaatgagtgagctaactcacattaattgcgttgcgctcacggcgcgccactttagtgaccaccctgacccatggcgtgcagtgcttcttaattaaagcagcacgacttcttcaagtccgccatgcccgaaggctacgtccaggagcgcaccatcttcttcaaggacgacggcaactacaagacccgcgccgaggtgaagttcgagggcgacaccctggtgaaccgcatcgagctgaagggcatcgacttcaaggaggacggcaacatcctggggcacaagctggagtacaactacaacagccacaacgtctatatcatggccgacaagcagaagaacggcatcaaggtgaacttcaagatccgccacaacatcgaggacggcagcgtgcagctcgccgaccactaccagcagaacacccccatcggcgacggccccgtgctgctgcccgacaaccactacctgagcacccagtccgccctgagcaaagaccccaacgagaagcgcgatcacatggtcctgctggagttcgtgaccgccgccgggatcactctcggcatggacgagctgtacaatga

(N-GFP is highlighted in blue. C-GFP is highlighted in purple.)

>pSSA2-GFP reading frame

atggtgagcaagggcgaggagctgttcaccggggtggtgcccatcctggtcgagctggacggcgacgtaaacggccacaagttcagcgtgtccggcgagggcgagggcgatgccacctacggcaagctgaccctgaagttcatctgcaccaccggcaagctgcccgtgccctggcccaccctcgtgaccaccctgacctacggcgtgcagtgcttcagccgctaccccgaccacatgaagcagcacgacttcttcaagtccgccatgcccgaaggctacgtccaggagcgcaccatcttcttcaaggacgacggcaactacaagacccgcgccgaggtgaagttcgagggcgacaccctggtgaaccgcatcgagctgaagggcatcgacttcaaggaggacggcaacatcctggggcacaagctggagtacaactacaacagccacaacgtctatatcatggccgacaagcagaagtgataaggatcccgggatcctaagcttgatgcatagcttgagtattctaacgcgtcacctaaatagcttggcgtaatcatggtcatagctgtttcctgtgtgaaattgttatccgctcacaattccacacaacatacgagccggaagcataaagtgtaaagcctggggtgcctaatgagtgagctaactcacattaattgcgttgcgctcacggcgcgccactttagtgaccaccctgacccatggcgtgcagtgcttcttaattaaagcagcacgacttcttcaagtccgccatgcccgaaggctacgtccaggagcgcaccatcttcttcaaggacgacggcaactacaagacccgcgccgaggtgaagttcgagggcgacaccctggtgaaccgcatcgagctgaagggcatcgacttcaaggaggacggcaacatcctggggcacaagctggagtacaactacaacagccacaacgtctatatcatggccgacaagcagaagaacggcatcaaggtgaacttcaagatccgccacaacatcgaggacggcagcgtgcagctcgccgaccactaccagcagaacacccccatcggcgacggccccgtgctgctgcccgacaaccactacctgagcacccagtccgccctgagcaaagaccccaacgagaagcgcgatcacatggtcctgctggagttcgtgaccgccgccgggatcactctcggcatggacgagctgtacaatga

(N-GFP is highlighted in blue. C-GFP is highlighted in purple.)

>pSSA3-GFP reading frame

atggtgagcaagggcgaggagctgttcaccggggtggtgcccatcctggtcgagctggacggcgacgtaaacggccacaagttcagcgtgtccggcgagggcgagggcgatgccacctacggcaagctgaccctgaagttcatctgcaccaccggcaagctgcccgtgccctggcccaccctcgtgaccaccctgacctacggcgtgcagtgcttcagccgctaccccgaccacatgaagcagcacgacttcttcaagtccgccatgcccgaaggctacgtccaggagcgcaccatcttctgataaggatcccgggatcctaagcttgatgcatagcttgagtattctaacgcgtcacctaaatagcttggcgtaatcatggtcatagctgtttcctgtgtgaaattgttatccgctcacaattccacacaacatacgagccggaagcataaagtgtaaagcctggggtgcctaatgagtgagctaactcacattaattgcgttgcgctcacggcgcgccactttagtgaccaccctgacccatggcgtgcagtgcttcttaattaaatggtgagcaagggcgaggagctgttcaccggggtggtgcccatcctggtcgagctggacggcgacgtaaacggccacaagttcagcgtgtccggcgagggcgagggcgatgccacctacggcaagctgaccctgaagttcatctgcaccaccggcaagctgcccgtgccctggcccaccctcgtgaccaccctgacctacggcgtgcagtgcttcagccgctaccccgaccacatgaagcagcacgacttcttcaagtccgccatgcccgaaggctacgtccaggagcgcaccatcttcttcaaggacgacggcaactacaagacccgcgccgaggtgaagttcgagggcgacaccctggtgaaccgcatcgagctgaagggcatcgacttcaaggaggacggcaacatcctggggcacaagctggagtacaactacaacagccacaacgtctatatcatggccgacaagcagaagaacggcatcaaggtgaacttcaagatccgccacaacatcgaggacggcagcgtgcagctcgccgaccactaccagcagaacacccccatcggcgacggccccgtgctgctgcccgacaaccactacctgagcacccagtccgccctgagcaaagaccccaacgagaagcgcgatcacatggtcctgctggagttcgtgaccgccgccgggatcactctcggcatggacgagctgtacaag

(N-GFP is highlighted in blue. C-GFP is highlighted in purple.)

>pSSA4-GFP reading frame

atggtgagcaagggcgaggagctgttcaccggggtggtgcccatcctggtcgagctggacggcgacgtaaacggccacaagttcagcgtgtccggcgagggcgagggcgatgccacctacggcaagctgaccctgaagttcatctgcaccaccggcaagctgcccgtgccctggcccaccctcgtgaccaccctgacctacggcgtgcagtgcttcagccgctaccccgaccacatgaagcagcacgacttcttcaagtccgccatgcccgaaggctacgtccaggagcgcaccatcttcttcaaggacgacggcaactacaagacccgcgccgaggtgaagttcgagggcgacaccctggtgaaccgcatcgagctgaagggcatcgacttcaaggaggacggcaacatcctggggcacaagctggagtacaactacaacagccacaacgtctatatcatggccgacaagcagaagtgataaggatcccgggatcctaagcttgatgcatagcttgagtattctaacgcgtcacctaaatagcttggcgtaatcatggtcatagctgtttcctgtgtgaaattgttatccgctcacaattccacacaacatacgagccggaagcataaagtgtaaagcctggggtgcctaatgagtgagctaactcacattaattgcgttgcgctcacggcgcgccactttagtgaccaccctgacccatggcgtgcagtgcttcttaattaaatggtgagcaagggcgaggagctgttcaccggggtggtgcccatcctggtcgagctggacggcgacgtaaacggccacaagttcagcgtgtccggcgagggcgagggcgatgccacctacggcaagctgaccctgaagttcatctgcaccaccggcaagctgcccgtgccctggcccaccctcgtgaccaccctgacctacggcgtgcagtgcttcagccgctaccccgaccacatgaagcagcacgacttcttcaagtccgccatgcccgaaggctacgtccaggagcgcaccatcttcttcaaggacgacggcaactacaagacccgcgccgaggtgaagttcgagggcgacaccctggtgaaccgcatcgagctgaagggcatcgacttcaaggaggacggcaacatcctggggcacaagctggagtacaactacaacagccacaacgtctatatcatggccgacaagcagaagaacggcatcaaggtgaacttcaagatccgccacaacatcgaggacggcagcgtgcagctcgccgaccactaccagcagaacacccccatcggcgacggccccgtgctgctgcccgacaaccactacctgagcacccagtccgccctgagcaaagaccccaacgagaagcgcgatcacatggtcctgctggagttcgtgaccgccgccgggatcactctcggcatggacgagctgtacaag

(N-GFP is highlighted in blue. C-GFP is highlighted in purple.)

>pSSA-Gaussia reading frame

ggagtcaaagttctgtttgccctgatctgcatcgctgtggccgaggccaagcccaccgagaacaacgaagacttcaacatcgtggccgtggccagcaacttcgcgaccacggatctcgatgctgaccgcgggaagttgcccggcaagaagctgccgctggaggtgctcaaagagatggaagccaatgcccggaaagctggctgcaccaggggctgtctgatctgcctgtcccacatcaagtgcacgcccaagatgaagaagttcatcccaggacgctgccacacctacgaaggcgacaaagagtccgcacagggcggcataggcgaggcgatcgtcgacattcctgagattcctgggttcaaggacttgtgataacgggatcctaagcttgatgcatagcttgagtattctaacgcgtcacctaaatagcttggcgtaatcatggtcatagctgtttcctgtgtgaaattgttatccgctcacaattccacacaacatacgagccggaagcataaagtgtaaagcctggggtgcctaatgagtgagctaactcacattaattgcgttgcgctcacggcgcgccactttagtgaccaccctgacccatggcgtgcagtgcttcttaattaatgcccggaaagctggctgcaccaggggctgtctgatctgcctgtcccacatcaagtgcacgcccaagatgaagaagttcatcccaggacgctgccacacctacgaaggcgacaaagagtccgcacagggcggcataggcgaggcgatcgtcgacattcctgagattcctgggttcaaggacttggagcccatggagcagttcatcgcacaggtcgatctgtgtgtggactgcacaactggctgcctcaaagggcttgccaacgtgcagtgttctgacctgctcaagaagtggctgccgcaacgctgtgcgacctttgccagcaagatccagggccaggtggacaagatcaagggggccggtggtgactaa

(N-Gau is highlighted in blue. C-Gau is highlighted in purple.)

>HDR target1-GFP reading frame

Atggtgagcaagggcgaggagctgttcaccggggtggtgcccatcctggtcgagctggacggcgacgtaaacggccacaagttcagcgtgtccggcgagggcgagggcgatgccacctacggcaagctgaccctgaagttcatctgcaccaccggcaagctgcccgtgccctggcccaccctcgtgaccaccctgacctacggcgtgcagtgcttcagccgctaccccgaccacatgaagcagcacgacttcttcaagtccgccatgcccgaaggctacgtccaggagcgcaccatcttctgataacgggatcctaagcttgatgcatagcttgagtattctaacgcgtcacctaaatagcttggcgtaatcatggtcatagctgtttcctgtgtgaaattgttatccgctcacaattccacacaacatacgagccggaagcataaagtgtaaagcctggggtgcctaatgagtgagctaactcacattaattgcgttgcgctcacggcgcgccactttagtgaccaccctgacccatggcgtgcagtgcttcttaattaattcaaggacgacggcaactacaagacccgcgccgaggtgaagttcgagggcgacaccctggtgaaccgcatcgagctgaagggcatcgacttcaaggaggacggcaacatcctggggcacaagctggagtacaactacaacagccacaacgtctatatcatggccgacaagcagaagaacggcatcaaggtgaacttcaagatccgccacaacatcgaggacggcagcgtgcagctcgccgaccactaccagcagaacacccccatcggcgacggccccgtgctgctgcccgacaaccactacctgagcacccagtccgccctgagcaaagaccccaacgagaagcgcgatcacatggtcctgctggagttcgtgaccgccgccgggatcactctcggcatggacgagctgtacaagtaa

(N-GFP is highlighted in blue. C-GFP is highlighted in purple.)

>HDR target2-GFP reading frame

atggtgagcaagggcgaggagctgttcaccggggtggtgcccatcctggtcgagctggacggcgacgtaaacggccacaagttcagcgtgtccggcgagggcgagggcgatgccacctacggcaagctgaccctgaagttcatctgcaccaccggcaagctgcccgtgccctggcccaccctcgtgaccaccctgacctacggcgtgcagtgcttcagccgctaccccgaccacatgaagcagcacgacttcttcaagtccgccatgcccgaaggctacgtccaggagcgcaccatcttcttcaaggacgacggcaactacaagacccgcgccgaggtgaagttcgagggcgacaccctggtgaaccgcatcgagctgaagggcatcgacttcaaggaggacggcaacatcctggggcacaagctggagtacaactacaacagccacaacgtctatatcatggccgacaagcagaagtgataacgggatcctaagcttgatgcatagcttgagtattctaacgcgtcacctaaatagcttggcgtaatcatggtcatagctgtttcctgtgtgaaattgttatccgctcacaattccacacaacatacgagccggaagcataaagtgtaaagcctggggtgcctaatgagtgagctaactcacattaattgcgttgcgctcacggcgcgccactttagtgaccaccctgacccatggcgtgcagtgcttcttaattaaaacggcatcaaggtgaacttcaagatccgccacaacatcgaggacggcagcgtgcagctcgccgaccactaccagcagaacacccccatcggcgacggccccgtgctgctgcccgacaaccactacctgagcacccagtccgccctgagcaaagaccccaacgagaagcgcgatcacatggtcctgctggagttcgtgaccgccgccgggatcactctcggcatggacgagctgtacaagtaa

(N-GFP is highlighted in blue. C-GFP is highlighted in purple.)

>HDR-GFP donor

tcgcgatgaataaatgaaagcttgcagatctgcgactctagaggatctgcgactctagaggatcataatcagccataccacatttgtagaggttttacttgctttaaaaaacctcccacacctccccctgaacctgaaacataaaatgaatgcaattgttgttgttaacttgtttattgcagcttataatggttacaaataaagcaatagcatcacaaatttcacaaataaagcatttttttcactgcattctagttgtggtttgtccaaactcatcaatgtatcttatcatgtctggatctgcgactctagaggatcataatcagccataccacatttgtagaggttttacttgctttaaaaaacctcccacacctccccctgaacctgaaacataaaatgaatgcaattgttgttgttaacttgtttattgcagcttataatggttacaaataaagcaatagcatcacaaatttcacaaataaagcatttttttcactgcattctagttgtggtttgtccaaactcatcaatgtatcttatcatgtctggatctgcgactctagaggatcataatcagccataccacatttgtagaggttttacttgctttaaaaaacctcccacacctccccctgaacctgaaacataaaatgaatgcaattgttgttgttaacttgtttattgcagcttataatggttacaaataaagcaatagcatcacaaatttcacaaataaagcatttttttcactgcattctagttgtggtttgtccaaactcatcaatgtatcttatcatgtctggatccccatcaagctgatccggaacccttaatgctagcaatcgatagtactaacatacgctctccatcaaaacaaaacgaaacaaaacaaactagcaaaataggctgtccccagtgcaagtgcaggtgccagaacatttctctatcgataggcgcgcctggattaattaatggaagtggaagtggaagtggaatggtgagcaagggcgaggagctgttcaccggggtggtgcccatcctggtcgagctggacggcgacgtaaacggccacaagttcagcgtgtccggcgagggcgagggcgatgccacctacggcaagctgaccctgaagttcatctgcaccaccggcaagctgcccgtgccctggcccaccctcgtgaccaccctgacctacggcgtgcagtgcttcagccgctaccccgaccacatgaagcagcacgacttcttcaagtccgccatgcccgaaggctacgtccaggagcgcaccatcttcttcaaggacgacggcaactacaagacccgcgccgaggtgaagttcgagggcgacaccctggtgaaccgcatcgagctgaagggcatcgacttcaaggaggacggcaacatcctggggcacaagctggagtacaactacaacagccacaacgtctatatcatggccgacaagcagaagaacggcatcaaggtgaacttcaagatccgccacaacatcgaggacggcagcgtgcagctcgccgaccactaccagcagaacacccccatcggcgacggccccgtgctgctgcccgacaaccactacctgagcacccagtccgccctgagcaaagaccccaacgagaagcgcgatcacatggtcctgctggagttcgtgaccgccgccgggatcactctcggcatggacgagctgtacaagtaa

(Stop sequence is highlighted in gray. GFP is highlighted in blue.)

>HDR-Gaussia reading frame

Ggagtcaaagttctgtttgccctgatctgcatcgctgtggccgaggccaagcccaccgagaacaacgaagacttcaacatcgtggccgtggccagcaacttcgcgaccacggatctcgatgctgaccgcgggaagttgcccggcaagaagctgccgctggaggtgctcaaagagatggaagccaatgcccggaaagctggctgcaccaggggctgtctgatctgcctgtcccacatcaagtgcacgcccaagatgaagaagttcatcccaggacgctgccacacctacgaaggcgacaaagagtccgcacagggcggcataggcgaggcgatcgtcgacattcctgagattcctgggttcaaggacttgtgataacgggatcctaagcttgatgcatagcttgagtattctaacgcgtcacctaaatagcttggcgtaatcatggtcatagctgtttcctgtgtgaaattgttatccgctcacaattccacacaacatacgagccggaagcataaagtgtaaagcctggggtgcctaatgagtgagctaactcacattaattgcgttgcgctcacggcgcgccactttagtgaccaccctgacccatggcgtgcagtgcttcttaattaagagcccatggagcagttcatcgcacaggtcgatctgtgtgtggactgcacaactggctgcctcaaagggcttgccaacgtgcagtgttctgacctgctcaagaagtggctgccgcaacgctgtgcgacctttgccagcaagatccagggccaggtggacaagatcaagggggccggtggtgactaa

(N-Gau is highlighted in blue. C-Gau is highlighted in purple.)

>HDR-Gaussia donor

tcgcgatgaataaatgaaagcttgcagatctgcgactctagaggatctgcgactctagaggatcataatcagccataccacatttgtagaggttttacttgctttaaaaaacctcccacacctccccctgaacctgaaacataaaatgaatgcaattgttgttgttaacttgtttattgcagcttataatggttacaaataaagcaatagcatcacaaatttcacaaataaagcatttttttcactgcattctagttgtggtttgtccaaactcatcaatgtatcttatcatgtctggatctgcgactctagaggatcataatcagccataccacatttgtagaggttttacttgctttaaaaaacctcccacacctccccctgaacctgaaacataaaatgaatgcaattgttgttgttaacttgtttattgcagcttataatggttacaaataaagcaatagcatcacaaatttcacaaataaagcatttttttcactgcattctagttgtggtttgtccaaactcatcaatgtatcttatcatgtctggatctgcgactctagaggatcataatcagccataccacatttgtagaggttttacttgctttaaaaaacctcccacacctccccctgaacctgaaacataaaatgaatgcaattgttgttgttaacttgtttattgcagcttataatggttacaaataaagcaatagcatcacaaatttcacaaataaagcatttttttcactgcattctagttgtggtttgtccaaactcatcaatgtatcttatcatgtctggatccccatcaagctgatccggaacccttaatgctagcaatcgatagtactaacatacgctctccatcaaaacaaaacgaaacaaaacaaactagcaaaataggctgtccccagtgcaagtgcaggtgccagaacatttctctatcgataggcgcgcctggattaattaatggaagtggaagtggaagtggaggagtcaaagttctgtttgccctgatctgcatcgctgtggccgaggccaagcccaccgagaacaacgaagacttcaacatcgtggccgtggccagcaacttcgcgaccacggatctcgatgctgaccgcgggaagttgcccggcaagaagctgccgctggaggtgctcaaagagatggaagccaatgcccggaaagctggctgcaccaggggctgtctgatctgcctgtcccacatcaagtgcacgcccaagatgaagaagttcatcccaggacgctgccacacctacgaaggcgacaaagagtccgcacagggcggcataggcgaggcgatcgtcgacattcctgagattcctgggttcaaggacttggagcccatggagcagttcatcgcacaggtcgatctgtgtgtggactgcacaactggctgcctcaaagggcttgccaacgtgcagtgttctgacctgctcaagaagtggctgccgcaacgctgtgcgacctttgccagcaagatccagggccaggtggacaagatcaagggggccggtggtgactaa

(Stop sequence is highlighted in gray. Gaussia is highlighted in blue.)

>NHEJ+1-GFP reading frame

atgtttagtgaccaccctgacccatggcgtgcagtgcttcagggtacctgtgagcaagggcgaggagctgttcaccggggtggtgcccatcctggtcgagctggacggcgacgtaaacggccacaagttcagcgtgtccggcgagggcgagggcgatgccacctacggcaagctgaccctgaagttcatctgcaccaccggcaagctgcccgtgccctggcccaccctcgtgaccaccctgacctacggcgtgcagtgcttcagccgctaccccgaccacatgaagcagcacgacttcttcaagtccgccatgcccgaaggctacgtccaggagcgcaccatcttcttcaaggacgacggcaactacaagacccgcgccgaggtgaagttcgagggcgacaccctggtgaaccgcatcgagctgaagggcatcgacttcaaggaggacggcaacatcctggggcacaagctggagtacaactacaacagccacaacgtctatatcatggccgacaagcagaagaacggcatcaaggtgaacttcaagatccgccacaacatcgaggacggcagcgtgcagctcgccgaccactaccagcagaacacccccatcggcgacggccccgtgctgctgcccgacaaccactacctgagcacccagtccgccctgagcaaagaccccaacgagaagcgcgatcacatggtcctgctggagttcgtgaccgccgccgggatcactctcggcatggacgagctgtacaagtga

> NHEJ+2-GFP reading frame

atgaatttagtgaccaccctgacccatggcgtgcagtgcttcagggtaccgtgagcaagggcgaggagctgttcaccggggtggtgcccatcctggtcgagctggacggcgacgtaaacggccacaagttcagcgtgtccggcgagggcgagggcgatgccacctacggcaagctgaccctgaagttcatctgcaccaccggcaagctgcccgtgccctggcccaccctcgtgaccaccctgacctacggcgtgcagtgcttcagccgctaccccgaccacatgaagcagcacgacttcttcaagtccgccatgcccgaaggctacgtccaggagcgcaccatcttcttcaaggacgacggcaactacaagacccgcgccgaggtgaagttcgagggcgacaccctggtgaaccgcatcgagctgaagggcatcgacttcaaggaggacggcaacatcctggggcacaagctggagtacaactacaacagccacaacgtctatatcatggccgacaagcagaagaacggcatcaaggtgaacttcaagatccgccacaacatcgaggacggcagcgtgcagctcgccgaccactaccagcagaacacccccatcggcgacggccccgtgctgctgcccgacaaccactacctgagcacccagtccgccctgagcaaagaccccaacgagaagcgcgatcacatggtcctgctggagttcgtgaccgccgccgggatcactctcggcatggacgagctgtacaagtga

>NHEJ Stop+1-GFP reading frame

atgtttagtgaccaccctgacccatggcgtgcagtgcttcagggtacctagtagtagcgtgagcaagggcgaggagctgttcaccggggtggtgcccatcctggtcgagctggacggcgacgtaaacggccacaagttcagcgtgtccggcgagggcgagggcgatgccacctacggcaagctgaccctgaagttcatctgcaccaccggcaagctgcccgtgccctggcccaccctcgtgaccaccctgacctacggcgtgcagtgcttcagccgctaccccgaccacatgaagcagcacgacttcttcaagtccgccatgcccgaaggctacgtccaggagcgcaccatcttcttcaaggacgacggcaactacaagacccgcgccgaggtgaagttcgagggcgacaccctggtgaaccgcatcgagctgaagggcatcgacttcaaggaggacggcaacatcctggggcacaagctggagtacaactacaacagccacaacgtctatatcatggccgacaagcagaagaacggcatcaaggtgaacttcaagatccgccacaacatcgaggacggcagcgtgcagctcgccgaccactaccagcagaacacccccatcggcgacggccccgtgctgctgcccgacaaccactacctgagcacccagtccgccctgagcaaagaccccaacgagaagcgcgatcacatggtcctgctggagttcgtgaccgccgccgggatcactctcggcatggacgagctgtacaagtga

>NHEJ-Gaussia reading frame

atgtttagtgaccaccctgacccatggcgtgcagtgcttcagggtacctagtagtagcggagtcaaagttctgtttgccctgatctgcatcgctgtggccgaggccaagcccaccgagaacaacgaagacttcaacatcgtggccgtggccagcaacttcgcgaccacggatctcgatgctgaccgcgggaagttgcccggcaagaagctgccgctggaggtgctcaaagagatggaagccaatgcccggaaagctggctgcaccaggggctgtctgatctgcctgtcccacatcaagtgcacgcccaagatgaagaagttcatcccaggacgctgccacacctacgaaggcgacaaagagtccgcacagggcggcataggcgaggcgatcgtcgacattcctgagattcctgggttcaaggacttggagcccatggagcagttcatcgcacaggtcgatctgtgtgtggactgcacaactggctgcctcaaagggcttgccaacgtgcagtgttctgacctgctcaagaagtggctgccgcaacgctgtgcgacctttgccagcaagatccagggccaggtggacaagatcaagggggccggtggtgactaa

>mTmG reading frame

actttagtgaccaccctgacccatggcgtgcagtgcttcgatatcgggcccatgggttgctgtttctccaagaccatggtgagcaagggagaggaggtcatcaaagagttcatgcgcttcaaggtgcgcatggagggctccatgaacggccacgagttcgagatcgagggcgagggcgagggccgcccctacgagggcacccagaccgccaagctgaaggtgaccaagggcggccccctgcccttcgcctgggacatcctgtccccccagttcatgtacggctccaaggcgtacgtgaagcaccccgccgacatccccgattacaagaagctgtccttccccgagggcttcaagtgggagcgcgtgatgaacttcgaggacggcggtctggtgaccgtgacccaggactcctccctgcaggacggcacgctgatctacaaggtgaagatgcgcggcaccaacttcccccccgacggccccgtaatgcagaagaagaccatgggctgggaggcctccaccgagcgcctgtacccccgcgacggcgtgctgaagggcgagatccaccaggccctgaagctgaaggacggcggccactacctggtggagttcaagaccatctacatggccaagaagcccgtgcaactgcccggctactactacgtggacaccaagctggacatcacctcccacaacgaggactacaccatcgtggaacagtacgagcgctccgagggccgccaccacctgttcctggggcatggcaccggcagcaccggcagcggcagctccggcaccgcctcctccgaggacaacaacatggccgtcatcaaagagttcatgcgcttcaaggtgcgcatggagggctccatgaacggccacgagttcgagatcgagggcgagggcgagggccgcccctacgagggcacccagaccgccaagctgaaggtgaccaagggcggccccctgcccttcgcctgggacatcctgtccccccagttcatgtacggctccaaggcgtacgtgaagcaccccgccgacatccccgattacaagaagctgtccttccccgagggcttcaagtgggagcgcgtgatgaacttcgaggacggcggtctggtgaccgtgacccaggactcctccctgcaggacggcacgctgatctacaaggtgaagatgcgcggcaccaacttcccccccgacggccccgtaatgcagaagaagaccatgggctgggaggcctccaccgagcgcctgtacccccgcgacggcgtgctgaagggcgagatccaccaggccctgaagctgaaggacggcggccgctacctggtggagttcaagaccatctacatggccaagaagcccgtgcaactgcccggctactactacgtggacaccaagctggacatcacctcccacaacgaggactacaccatcgtggaacagtacgagcgctccgagggccgccaccacctgttcctgtacggcatggacgagctgtacaagtaattaattgactcgagactagtgaattcattcgagggatctttgtgaaggaaccttacttctgtggtgtgacataattggacaaactacctacagagatttaaagctctaaggtaaatataaaatttttaagtgtataatgtgttaaactactgattctaattgtttgtgtattttagattccaacctatggaactgatgaatgggagcagtggtggaatgcctttaatgaggaaaacctgttttgctcagaagaaatgccatctagtgatgatgaggctactgctgactctcaacattctactcctccaaaaaagaagagaaaggtagaagaccccaaggactttccttcagaattgctaagttttttgagtcatgctgtgtttagtaatagaactcttgcttgctttgctatttacaccacaaaggaaaaagctgcactgctatacaagaaaattatggaaaaatattctgtaacctttataagtaggcataacagttataatcataacatactgttttttcttactccacacaggcatagagtgtctgctattaataactatgctcaaaaattgtgtacctttagctttttaatttgtaaaggggttaataaggaatatttgatgtatagtgccttgactagagatcataatcagccataccacatttgtagaggttttacttgctttaaaaaacctcccacacctccccctgaacctgaaacataaaatgaatgcaattgttgttgttaacttgtttattgcagcttataatggttacaaataaagcaatagcatcacaaatttcacaaataaagcatttttttcactgcattctagttgtggtttgtccaaactcatcaatgtatcttatcatgtctggatctgacatggtaagtaagcttgggctgcacgcgtcgagggactttagtgaccaccctgacccatggcgtgcagtgcttccggtacctccggatcatcaccgcggatgggttgctgtttctccaagaccgcagcgaagggagaagccgccgcggagaggcctggggaggcggctgtggcctcgtcgccttccaaagcgaacggacaggagaatggccacgtgaaggtaggatccgtgagcaagggcgaggagctgttcaccggggtggtgcccatcctggtcgagctggacggcgacgtaaacggccacaagttcagcgtgtccggcgagggcgagggcgatgccacctacggcaagctgaccctgaagttcatctgcaccaccggcaagctgcccgtgccctggcccaccctcgtgaccaccctgacctacggcgtgcagtgcttcagccgctaccccgaccacatgaagcagcacgacttcttcaagtccgccatgcccgaaggctacgtccaggagcgcaccatcttcttcaaggacgacggcaactacaagacccgcgccgaggtgaagttcgagggcgacaccctggtgaaccgcatcgagctgaagggcatcgacttcaaggaggacggcaacatcctggggcacaagctggagtacaactacaacagccacaacgtctatatcatggccgacaagcagaagaacggcatcaaggtgaacttcaagatccgccacaacatcgaggacggcagcgtgcagctcgccgaccactaccagcagaacacccccatcggcgacggccccgtgctgctgcccgacaaccactacctgagcacccagtccgccctgagcaaagaccccaacgagaagcgcgatcacatggtcctgctggagttcgtgaccgccgccgggatcactctcggcatggacgagctgtacaagtaa

(Tomato is highlighted in red. GFP is highlighted in green.)

**Note 2. Supplementary sequences of amino acids**

(* in following texts indicate stop codon.)

FCR reporter plasmid (mutant is highlighted in red.)

MVSKGEELFTGVVPILVELDGDVNGHKFSVSGEGEGDATYGKLTLKFICTTGKLPVPWPTLVTTLTHGVQCFSRYPDHMKQHDFFKSAMPEGYVQERTIFFKDDGNYKTRAEVKFEGDTLVNRIELKGIDFKEDGNILGHKLEYNYNSHNVYIMADKQKNGIKVNFKIRHNIEDGSVQLADHYQQNTPIGDGPVLLPDNHYLSTQSKLSKDPNEKRDHMVLLEFVTAAGITLGMDELYK*

PSSA reporter plasmid (repeat is highlighted in blue.)

PSSA1-GFP:

N-GFP: MVSKGEELFTGVVPILVELDGDVNGHKFSVSGEGEGDATYGKLTLKFICTTGKLPVPWPTLVTTLTYGVQCFSRYPDHMKQHDFFKSAMPEGYVQERTIF*

C-GFP:

QHDFFKSAMPEGYVQERTIFFKDDGNYKTRAEVKFEGDTLVNRIELKGIDFKEDGNILGHKLEYNYNSHNVYIMADKQKNGIKVNFKIRHNIEDGSVQLADHYQQNTPIGDGPVLLPDNHYLSTQSALSKDPNEKRDHMVLLEFVTAAGITLGMDELY*

PSSA2-GFP:

N-GFP:

MVSKGEELFTGVVPILVELDGDVNGHKFSVSGEGEGDATYGKLTLKFICTTGKLPVPWPTLVTTLTYGVQCFSRYPDHMKQHDFFKSAMPEGYVQERTIFFKDDGNYKTRAEVKFEGDTLVNRIELKGIDFKEDGNILGHKLEYNYNSHNVYIMADKQK*

C-GFP:

QHDFFKSAMPEGYVQERTIFFKDDGNYKTRAEVKFEGDTLVNRIELKGIDFKEDGNILGHKLEYNYNSHNVYIMADKQKNGIKVNFKIRHNIEDGSVQLADHYQQNTPIGDGPVLLPDNHYLSTQSALSKDPNEKRDHMVLLEFVTAAGITLGMDELY*

PSSA3-GFP:

N-GFP:

MVSKGEELFTGVVPILVELDGDVNGHKFSVSGEGEGDATYGKLTLKFICTTGKLPVPWPTLVTTLTYGVQCFSRYPDHMKQHDFFKSAMPEGYVQERTIF*

C-GFP:

MVSKGEELFTGVVPILVELDGDVNGHKFSVSGEGEGDATYGKLTLKFICTTGKLPVPWPTLVTTLTYGVQCFSRYPDHMKQHDFFKSAMPEGYVQERTIFFKDDGNYKTRAEVKFEGDTLVNRIELKGIDFKEDGNILGHKLEYNYNSHNVYIMADKQKNGIKVNFKIRHNIEDGSVQLADHYQQNTPIGDGPVLLPDNHYLSTQSALSKDPNEKRDHMVLLEFVTAAGITLGMDELYK*

PSSA4-GFP:

N-GFP:

MVSKGEELFTGVVPILVELDGDVNGHKFSVSGEGEGDATYGKLTLKFICTTGKLPVPWPTLVTTLTYGVQCFSRYPDHMKQHDFFKSAMPEGYVQERTIFFKDDGNYKTRAEVKFEGDTLVNRIELKGIDFKEDGNILGHKLEYNYNSHNVYIMADKQK*

C-GFP:

MVSKGEELFTGVVPILVELDGDVNGHKFSVSGEGEGDATYGKLTLKFICTTGKLPVPWPTLVTTLTYGVQCFSRYPDHMKQHDFFKSAMPEGYVQERTIFFKDDGNYKTRAEVKFEGDTLVNRIELKGIDFKEDGNILGHKLEYNYNSHNVYIMADKQKNGIKVNFKIRHNIEDGSVQLADHYQQNTPIGDGPVLLPDNHYLSTQSALSKDPNEKRDHMVLLEFVTAAGITLGMDELYK*

PSSA-Gaussia:

N-Gau:

MGVKVLFALICIAVAEAKPTENNEDFNIVAVASNFATTDLDADRGKLPGKKLPLEVLKEMEANARKAGCTRGCLICLSHIKCTPKMKKFIPGRCHTYEGDKESAQGGIGEAIVDIPEIPGFKDL*

C-Gau:

CPESWLHQGLSDLPVPHQVHAQDEEVHPRTLPHLRRRQRVRTGRHRRGDRRHS*DSWVQGLGAHGAVHRTGRSVCGLHNWLPQRACQRAVF*PAQEVAAATLCDLCQQDPGPGGQDQGGRW*L

HDR reporter plasmid

HDR1-GFP:

N-GFP:

MVSKGEELFTGVVPILVELDGDVNGHKFSVSGEGEGDATYGKLTLKFICTTGKLPVPWPTLVTTLTYGVQCFSRYPDHMKQHDFFKSAMPEGYVQERTIF*

C-GFP:

FKDDGNYKTRAEVKFEGDTLVNRIELKGIDFKEDGNILGHKLEYNYNSHNVYIMADKQKNGIKVNFKIRHNIEDGSVQLADHYQQNTPIGDGPVLLPDNHYLSTQSALSKDPNEKRDHMVLLEFVTAAGITLGMDELYK*

HDR2-GFP:

N-GFP:

MVSKGEELFTGVVPILVELDGDVNGHKFSVSGEGEGDATYGKLTLKFICTTGKLPVPWPTLVTTLTYGVQCFSRYPDHMKQHDFFKSAMPEGYVQERTIFFKDDGNYKTRAEVKFEGDTLVNRIELKGIDFKEDGNILGHKLEYNYNSHNVYIMADKQK*

C-GFP:

NGIKVNFKIRHNIEDGSVQLADHYQQNTPIGDGPVLLPDNHYLSTQSALSKDPNEKRDHMVLLEFVTAAGITLGMDELY*

HDR-Gaussia:

N-Gau:

MGVKVLFALICIAVAEAKPTENNEDFNIVAVASNFATTDLDADRGKLPGKKLPLEVLKEMEANARKAGCTRGCLICLSHIKCTPKMKKFIPGRCHTYEGDKESAQGGIGEAIVDIPEIPGFKDL*

C-Gau:

EPMEQFIAQVDLCVDCTTGCLKGLANVQCSDLLKKWLPQRCATFASKIQGQVDKIKGAGGD*

NHEJ reporter plasmid (target is highlighted in yellow):

NHEJ+1-GFP:

MFSDHPDPWRAVLQGTCEQGRGAVHRGGAHPGRAGRRRKRPQVQRVRRGRGRCHLRQADPEVHLHHRQAARALAHPRDHPDLRRAVLQPLPRPHEAARLLQVRHARRLRPGAHHLLQGRRQLQDPRRGEVRGRHPGEPHRAEGHRLQGGRQHPGAQAGVQLQQPQRLYHGRQAEERHQGELQDPPQHRGRQRAARRPLPAEHPHRRRPRAAARQPLPEHPVRPEQRPQREARSHGPAGVRDRRRDHSRHGRAVQV

NHEJ+2-GFP:

MNLVTTLTHGVQCFRVP*ARARSCSPGWCPSWSSWTAT*TATSSACPARARAMPPTAS*P*SSSAPPASCPCPGPPS*PP*PTACSASAATPTT*SSTTSSSPPCPKATSRSAPSSSRTTATTRPAPR*SSRATPW*TASS*RASTSRRTATSWGTSWSTTTTATTSISWPTSRRTASR*TSRSATTSRTAACSSPTTTSRTPPSATAPCCCPTTTT*APSPP*AKTPTRSAITWSCWSS*PPPGSLSAWTSCTS

NHEJ Stop+1-GFP:

MFSDHPDPWRAVLQGT***REQGRGAVHRGGAHPGRAGRRRKRPQVQRVRRGRGRCHLRQADPEVHLHHRQAARALAHPRDHPDLRRAVLQPLPRPHEAARLLQVRHARRLRPGAHHLLQGRRQLQDPRRGEVRGRHPGEPHRAEGHRLQGGRQHPGAQAGVQLQQPQRLYHGRQAEERHQGELQDPPQHRGRQRAARRPLPAEHPHRRRPRAAARQPLPEHPVRPEQRPQREARSHGPAGVRDRRRDHSRHGRAVQV

NHEJ-Gaussia:

MFSDHPDPWRAVLQGT***RSQSSVCPDLHRCGRGQAHREQRRLQHRGRGQQLRDHGSRC*PREVARQEAAAGGAQRDGSQCPESWLHQGLSDLPVPHQVHAQDEEVHPRTLPHLRRRQRVRTGRHRRGDRRHS*DSWVQGLGAHGAVHRTGRSVCGLHNWLPQRACQRAVF*PAQEVAAATLCDLCQQDPGPGGQDQGGRW*L

mTmG reporter plasmid (target is highlighted in yellow, Tomato is highlighted in red, GFP is highlighted in green):

AL**PP*PMACSASISGMGCCFSKTMVSKGEEVIKEFMRFKVRMEGSMNGHEFEIEGEGEGRPYEGTQTAKLKVTKGGPLPFAWDILSPQFMYGSKAYVKHPADIPDYKKLSFPEGFKWERVMNFEDGGLVTVTQDSSLQDGTLIYKVKMRGTNFPPDGPVMQKKTMGWEASTERLYPRDGVLKGEIHQALKLKDGGHYLVEFKTIYMAKKPVQLPGYYYVDTKLDITSHNEDYTIVEQYERSEGRHHLFLGHGTGSTGSGSSGTASSEDNNMAVIKEFMRFKVRMEGSMNGHEFEIEGEGEGRPYEGTQTAKLKVTKGGPLPFAWDILSPQFMYGSKAYVKHPADIPDYKKLSFPEGFKWERVMNFEDGGLVTVTQDSSLQDGTLIYKVKMRGTNFPPDGPVMQKKTMGWEASTERLYPRDGVLKGEIHQALKLKDGGRYLVEFKTIYMAKKPVQLPGYYYVDTKLDITSHNEDYTIVEQYERSEGRHHLFLYGMDELYK*LIDSRLVNSFEGSL*RNLTSVV*HNWTNYLQRFKALR*I*NF*VYNVLNY*F*LFVYFRFQPMELMNGSSGGMPLMRKTCFAQKKCHLVMMRLLLTLNILLLQKRRER*KTPRTFLQNC*VF*VMLCLVIELLLALLFTPQRKKLHCYTRKLWKNIL*PL*VGITVIIITYCFFLLHTGIECLLLITMLKNCVPLAF*FVKGLIRNI*CIVP*LEIIISHTTFVEVLLALKNLPHLPLNLKHKMNAIVVVNLFIAAYNGYK*SNSITNFTNKAFFSLHSSCGLSKLINVSYHVWI*HGK*AWAARVEGL**PP*PMACSASGTSGSSPRMGCCFSKTAAKGEAAAERPGEAAVASSPSKANGQENGHVKVGSVSKGEELFTGVVPILVELDGDVNGHKFSVSGEGEGDATYGKLTLKFICTTGKLPVPWPTLVTTLTYGVQCFSRYPDHMKQHDFFKSAMPEGYVQERTIFFKDDGNYKTRAEVKFEGDTLVNRIELKGIDFKEDGNILGHKLEYNYNSHNVYIMADKQKNGIKVNFKIRHNIEDGSVQLADHYQQNTPIGDGPVLLPDNHYLSTQSALSKDPNEKRDHMVLLEFVTAAGITLGMDELYK*

**Table S1. Summary of gene editing reporter assays**

| Reporter Assay | Probed Repair Events | Target Cleavage By | Target Change  Flexibility | Reporter Delivery | Additional Preparation | Reporter Gene | Application |
| --- | --- | --- | --- | --- | --- | --- | --- |
| DR-GFP | HDR | I-*Sce*I or SpCas9 | No | Stable integration or  transfection | N/A | Fluorescent protein | Study mechanism of HDR |
| TLR | NHEJ/HDR | I-*Sce*I or SpCas9 | No | Stable integration | Sort mCherry negative cells | Fluorescent protein | Evaluate efficiency/outcome;  Isolate edited cells;  Study the DNA repair choices |
| EJ7-GFP | NHEJ | SpCas9 | Yes | Stable integration | N/A | Fluorescent protein | Study mechanism of NHEJ |
| DRR | NHEJ/HDR | I-*Sce*I | No | Stable integration | Monoclone selection | Fluorescent protein | Study mechanism of NHEJ and HDR |
| CDDR | NHEJ/HDR | SpCas9 | No | Stable integration | Monoclone selection | Fluorescent protein | Study mechanism of NHEJ and HDR |
| CAT-R | N/A | SpCas9 | No | Stable integration | Sort mCherry and GFP positive cells | Fluorescent protein | Probing small indels, large deletions and error-free repair;  Chemical and genetic screens |
| SSA reporter and HDR reporter | SSA/HDR | SpCas9 | Yes | Transfection | No | Fluorescent protein | Evaluate efficiency/outcome |
| FCR* | HDR | SpCas9/SaCas9/FnCpf1 | No | Transfection | No | Fluorescent protein | Evaluate the efficiency and outcome |
| NHEJ* | NHEJ | SpCas9/SaCas9/FnCpf1 | Yes | Transfection | No | Fluorescent protein/Luciferase | Study mechanism of NHEJ |
| HDR* | HDR | SpCas9/SaCas9/FnCpf1 | Yes | Transfection | No | Fluorescent protein/Luciferase | Evaluate efficiency/outcome; |
| pSSA* | SSA | SpCas9/SaCas9/FnCpf1 | Yes | Transfection | No | Fluorescent protein/Luciferase | Evaluate efficiency/outcome;  Isolate edited cells; |
| mTmG* | NHEJ | SpCas9/SaCas9/FnCpf1 | Yes | Transfection | No | Fluorescent protein | Manipulation tool for site-specific  genome editing |

*: reporter assays used in this study

Reference:

[1] Vriend, L.E., Jasin, M. and Krawczyk, P.M. (2014). Assaying break and nick-induced homologous recombination in mammalian cells using the DR-GFP reporter and Cas9 nucleases. Methods Enzymol 546, 175-91.

[2] Pierce, A.J., Johnson, R.D., Thompson, L.H. and Jasin, M. (1999). XRCC3 promotes homology-directed repair of DNA damage in mammalian cells. Genes Dev 13, 2633-8.

[3] Certo, M.T., Ryu, B.Y., Annis, J.E., Garibov, M., Jarjour, J., Rawlings, D.J. and Scharenberg, A.M. (2011). Tracking genome engineering outcome at individual DNA breakpoints. Nat Methods 8, 671-6.

[4] Metzger, M.J. and Certo, M.T. (2014). Design and analysis of site-specific single-strand nicking endonucleases for gene correction. Methods Mol Biol 1114, 237-44.

[5] Chu, V.T., Weber, T., Wefers, B., Wurst, W., Sander, S., Rajewsky, K. and Kuhn, R. (2015). Increasing the efficiency of homology-directed repair for CRISPR-Cas9-induced precise gene editing in mammalian cells. Nat Biotechnol 33, 543-8.

[6] Olivieri, M. et al. (2020). A Genetic Map of the Response to DNA Damage in Human Cells. Cell 182, 481-496 e21.

[7] Bhargava, R., Sandhu, M., Muk, S., Lee, G., Vaidehi, N. and Stark, J.M. (2018). C-NHEJ without indels is robust and requires synergistic function of distinct XLF domains. Nat Commun 9, 2484.

[8] Arnoult, N. et al. (2017). Regulation of DNA repair pathway choice in S and G2 phases by the NHEJ inhibitor CYREN. Nature 549, 548-552.

[9] Eki, R., She, J., Parlak, M., Benamar, M., Du, K.P., Kumar, P. and Abbas, T. (2020). A robust CRISPR-Cas9-based fluorescent reporter assay for the detection and quantification of DNA double-strand break repair. Nucleic Acids Res 48, e126.

[10] Roidos, P. et al. (2020). A scalable CRISPR/Cas9-based fluorescent reporter assay to study DNA double-strand break repair choice. Nat Commun 11, 4077.

[11] Yang, Y., Liu, S., Cheng, Y., Nie, L., Lv, C., Wang, G., Zhang, Y. and Hao, L. (2016). Highly Efficient and Rapid Detection of the Cleavage Activity of Cas9/gRNA via a Fluorescent Reporter. Appl Biochem Biotechnol 180, 655-667.

[12] Richardson, C.D., Ray, G.J., DeWitt, M.A., Curie, G.L. and Corn, J.E. (2016). Enhancing homology-directed genome editing by catalytically active and inactive CRISPR-Cas9 using asymmetric donor DNA. Nat Biotechnol 34, 339-44.

[13] Lu, J. et al. (2018). Multimode drug inducible CRISPR/Cas9 devices for transcriptional activation and genome editing. Nucleic Acids Res 46, e25.

[14] Zhao, C. et al. (2018). HIT-Cas9: A CRISPR/Cas9 Genome-Editing Device under Tight and Effective Drug Control. Mol Ther Nucleic Acids 13, 208-219.

[15] Keimling, M. et al. (2012). The power of DNA double-strand break (DSB) repair testing to predict breast cancer susceptibility. FASEB J 26, 2094-104.

[16] Yang, F. et al. (2017). CRISPR/Cas9-loxP-Mediated Gene Editing as a Novel Site-Specific Genetic Manipulation Tool. Mol Ther Nucleic Acids 7, 378-386.

**Table S2. Primers used in this study**

| Primer | Sequence |
| --- | --- |
| BFP sgRNA-SpCas9-F | cacc gctgaagcactgcacgccat |
| BFP sgRNA-SpCas9-R | aaac atggcgtgcagtgcttcagc |
| BFP sgRNA-SaCas9-F | cacc gaagcactgcacgccatgggt |
| BFP sgRNA-SaCas9-R | taac acccatggcgtgcagtgcttc |
| BFP crRNA-FnCpf1-F | caccgtaatttctactgttgtagatgtgaccaccctgacccatggcgtttttat |
| BFP crRNA-FnCpf1-R | cgataaaaacgccatgggtcagggtggtcacatctacaacagtagaaattac |
| AAVS1 sgRNA-SpCas9-F | cacc ggggccactagggacaggat |
| AAVS1 sgRNA-SpCas9-R | aaac atcctgtccctagtggcccc |
| TIDE-AAVS1-F | cctatgtccacttcaggacagc |
| TIDE-AAVS1-R | cctgccaagctctccctcccag |
| TIDE-BFP-F | gacgtaaacggccacaagttc |
| TIDE-BFP-R | ctcaggtagtggttgtcggg |
| GFP-F | cggggtacc atggtgagcaagggcgaggag |
| GFP-R | cgcggatcc ttacttgtacagctcgtccatgccgag |
| N-GFP-R-477bp | cgggatcc ttatcacttctgcttgtcggccatgat |
| N-GFP-R-300bp | cgggatcc ttatcagaagatggtgcgctcctggac |
| C-GFP-F-478bp | ttggcgcgcc agcagcacgacttcttcaagtc |
| C-GFP-F-417bp | ccttaattaa ttcaaggacgacggcaactac |
| C-GFP-F-240bp | ccttaattaa aacggcatcaaggtgaacttc |
| Gaussia-F | gctctaga gccaccatgggagtcaaagttctgtttgc |
| Gaussia-R | aacctgcagg ttagtcaccaccggcccccttg |
| N-Gaussia-R | cgggatcc cgttatcacaagtccttgaacccaggaatc |
| HDR-C-Gaussia-F | ccttaattaa gagcccatggagcagttcatc |
| pSSA-C-Gaussia-F | ccttaattaa tgcccggaaagctggctgcac |
